# Lasting effects of early exposure to temperature on the gonadal transcriptome at the time of sex differentiation in the European sea bass, a fish with mixed genetic and environmental sex determination

**DOI:** 10.1186/s12864-015-1862-0

**Published:** 2015-09-04

**Authors:** Noelia Díaz, Francesc Piferrer

**Affiliations:** Institut de Ciències del Mar, Consejo Superior de Investigaciones Científicas (CSIC), Passeig Marítim, 37-49, 08003 Barcelona, Spain; Present address: Max Planck Institute for Molecular Biomedicine, 48149 Münster, Germany

**Keywords:** Gonadal development, Temperature, Epigenetics, Sex ratio

## Abstract

**Background:**

Sex in fish is plastic and in several species can be influenced by environmental factors. In sensitive species, elevated temperatures have a masculinizing effect. Previous studies on the effects of temperature on gene expression have been restricted to a few cognate genes, mostly related to testis or ovarian development, and analyzed in gonads once they had completed the process of sex differentiation. However, studies on the effect of temperature at the whole gonadal transcriptomic level are scarce in fish and, in addition, temperature effects at the time of sex differentiation at the transcriptomic level are also unknown. Here, we used the European sea bass, a gonochoristic teleost with a polygenic sex determination system influenced by temperature, and exposed larvae to elevated temperature during the period of early gonad formation. Transcriptomic analysis of the gonads was carried out about three months after the end of temperature exposure, shortly after the beginning of the process of sex differentiation.

**Results:**

Elevated temperature doubled the number of males with respect to untreated controls. Transcriptomic analysis of early differentiating female gonads showed how heat caused: 1) an up-regulation of genes related to cholesterol transport (*star*), the stress response (*nr3c1*) and testis differentiation (*amh*, *dmrt*, etc.), 2) a decrease in the expression of genes related to ovarian differentiation such as *cyp19a1a*, and 3) an increase in the expression of several genes related to epigenetic regulatory mechanisms (*hdac11*, *dicer1*, *ehmt2*, *jarid2a*, *pcgf2*, *suz12*, *mettl22*).

**Conclusions:**

Taken together, the results of this study contribute to the understanding of how the early environment sets permanent changes that result in long-lasting consequences, in this case in the sexual phenotype. Results also show the usefulness of comparing the effects of heat on the behavior of cognate genes related to sex differentiation as well as that of genes involved in establishing and maintaining cell identity through epigenetic mechanisms.

**Electronic supplementary material:**

The online version of this article (doi:10.1186/s12864-015-1862-0) contains supplementary material, which is available to authorized users.

## Background

Identifying environmental cues and their perception and transduction mechanisms is a central focus of research in developmental biology within an ecological context [1]. Changes in environmental variables can have profound influences on differentiation, growth and reproduction in many organisms [2]. Temperature is the main abiotic factor that affects many biological functions at different levels of organization by changing the rates of chemical reactions and physiological processes, or by changing the three-dimensional shapes of biomolecules [3, 4].

Fish exhibit enormous diversity in their morphology, in habitat occupancy, and in their biology [5]. This diversity is also remarkable as regards to their reproductive strategies including sex determination and differentiation [6], two processes that contribute to the establishment of the sex ratio, a crucial parameter for population viability and for the continuation of all species with sexual reproduction. The sex ratio can be affected by environmental factors, mainly by temperature [7]. Among vertebrates with genetic sex determination (GSD) master sex determining genes are not conserved and eight genes with such a function have been identified so far: *Sry* in mammals, *DMRT-1* in birds, *DM-W* in *Xenopus laevis*, and *dmy*, *amhr2*, *amhy*, *sdy* and *gsdf* in fish [8, 9]. On the other hand, genes involved in the sex differentiation process (SD; [10, 11]) are fairly conserved in structure and dimorphic expression from fish to mammals [12].

Genes involved in testis differentiation include *dmrt1, dax1, sox9, arb, amh, cyp11b* [13–17], *sox9a2, tbx1a and tbx1b* [18]; whereas genes involved in ovarian differentiation include *cyp19a1a, foxl2, er, fst* [19–21, 17], *hsd3b* and *star* [18]. However, the order of expression and interactions among these genes may change between groups [22] or depending on environmental conditions [23]. In any case, estrogens are essential for proper ovarian differentiation in all non-mammalian vertebrates [24]. Thus, *cyp19a1a,* the gene that codes for aromatase, the enzyme that catalyzes the irreversible conversion of androgens into estrogens, is a major player in vertebrate SD and crucial for the establishment of the sex ratio. Aromatase gene expression is susceptible to environmental temperature influences. Therefore, and regardless of the sex ratio response pattern to temperature [25, 26], in reptiles and fish, the two types of vertebrates with temperature-dependent sex determination (TSD), the effects of environmental temperature on sex ratios are mediated by changes in *cyp19a1a* expression. In all fish species analyzed so far [26] more males are produced with increasing temperatures and *cyp19a1a* is always inhibited at male-producing temperatures and stimulated at female-producing temperatures [27], In general, elevated temperatures increase the expression of male-related genes such as *amh, dmrt1* or *arb* after the SD period, while decreasing the expression of female-related genes in addition to *cyp19a1a*, such as *esr1, esr2, erb1, fshr* or *foxl2* (see Additional file 1: Table S1 for a summary on general thermal effects on gene expression).

Effects of temperature on fish sex ratios are more pronounced if animals are exposed to elevated temperatures during early development. However, the number of genes known to be affected is limited and the metabolic and signaling pathways affected are essentially unknown. In this regard, several studies have explored the effects of cold and cold acclimation [2, 28] but focusing on tissues other than the gonads, such as liver, skeletal white muscle and gills [29–39]; brain [32, 39, 40] or heart [29, 32, 41]. Unfortunately, comparative transcriptomic studies on the effect of heat on the gonads are essentially limited to just one study with the pejerrey, *Odontesthes bonariensis*, a fish with TSD (Additional file 1: Table S1), and carried out on juvenile or adult mature gonads, not with differentiating gonads. Thus, it is difficult to ascertain whether observed altered patterns of gene expression are the cause or the consequence of a given gonadal phenotype resulting from exposure to elevated temperature.

The European sea bass is a eurythermal marine teleost able to live between 8 and 27 °C and a gonochoristic species with a polygenic system of sex determination [42], where genetics and temperature contribute essentially equally to sex ratios [43]. Recent studies in the sea bass have shown that *cyp19a1a* and *cyp11b* are good markers of female and male sex differentiation, respectively [17]. In all fish species studied so far, high temperatures cause masculinization. This is also the case of the European sea bass, where elevated temperatures during the thermosensitive period (TSP), which is located 0–60 dph, result in masculinization of about 50 % of the fish that otherwise, under more natural temperatures, would have developed into females, as assessed by sex ratio analysis [44–48]. Recently, we discovered that the effects of early temperature include hypermethylation of the *cyp19a1a* promoter in one-year-old juvenile females, with concomitant suppression of *cyp19a1a* expression and resulting in male instead of female development, the first evidence of an epigenetic link between environmental temperature and sex ratios in vertebrates [49].

We were interested in understanding the underlying mechanisms responsible for this masculinization at the time when the differentiating gonad is being affected. Therefore, instead of sampling one-year-old juveniles, as done before by us and others, in which gonads have already completed the process of sex differentiation, and where it is difficult to ascertain whether observed transcriptomic profiles and epigenetic changes are the cause or the consequence of that process, we sampled fish at 170 dph, i.e., 110 days after the end of temperature treatment but shortly after the start of sex differentiation (150 dph). Thus, the objective was to gain a better understanding of the genes and the pathways involved in sex differentiation that are directly affected by temperature at the time of sex differentiation.

## Results

### Growth, body indices and sex ratios

Because of the differences in rearing temperature between 20 and 60 dph, fish from the HT group were significantly (*P* < 0.05) larger than those of the LT group at 170 dph in both SL and BW (Table 1). Growth differences between groups had disappeared by 332 dph. Sexual growth dimorphism, in favor of females (*P* < 0.01), was present only in the LT group (Table 2). As observed in our previous studies survival was unrelated to temperature treatment (the difference between low and high temperature was only of 4 °C and both temperatures were within the range of natural temperature experienced by the European sea bass in the wild). Survival was ~60 % to the end of the larval stage and ~90 % thereafter.

**Table 1:** Growth of European sea bass juveniles at 170 days post hatch, classified according to treatment and *cyp19a1a* expression levels, as shown in Fig. 1a

		Low *cyp19a1a* expressors			High *cyp19a1a* expressors	
Treatment	N	Length (cm)	Weight (g)	N	Length (cm)	Weight (g)
LT	10	9.25 ± 0.196^a^	13.16 ± 0.967^a^	6	9.33 ± 0.061^a^	13.53 ± 0.581^a^
HT	9	9.86 ± 0.109^b^	17.41 ± 0.877^b^	7	10.28 ± 0.495^b^	19.35 ± 2.955^b^

Data as mean ± SEM

LT low temperature, HT high temperature, N sample size

Different letters indicate significant (*P* < 0.05) differences between treatments

**Table 2:** Growth of European sea bass juveniles at 332 days post hatch, classified according to treatment and sex

		Females			Males	
Treatment	N	Length (cm)	Weight (g)	N	Length (cm)	Weight (g)
LT	40	12.45 ± 0.180^a**^	33.16 ± 1.560^a**^	26	11.66 ± 0.223^a^	27.22 ± 1.867^a^
HT	16	12.22 ± 0.171^a^	33.38 ± 3.044^a^	60	12.36 ± 0.171^a^	33.79 ± 1.544^a^

Data as mean ± SEM

LT low temperature, HT high temperature, N sample size

The same superscript a indicates lack of significant (*P* > 0.05) differences between treatments. Asterisks indicate statistical differences (*P* < 0.01) between females and males within the same treatment group, i.e., sexual growth dimorphism

**Fig. 1:**
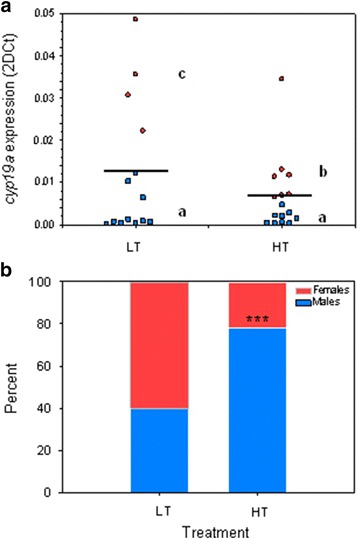
**a** Individual gonadal aromatase (*cyp19a1a*) expression levels (2DCt) as assessed by qRT-PCR in the low (LT; *N* = 16) and high (HT; *N* = 16) temperature groups at 170 dph. Blue squares and red circles correspond to individual fish with low (putative future males) and high (putative future females) *cyp19a1a* levels, respectively. The horizontal line marks mean expression for each experimental group. The *cyp19a1a* expression of one of the putative females in the LT group (red circles) was much higher than the rest and it has been deliberately omitted from the graph for clarity purposes. Different letters indicate significant (ANOVA; *P* < 0.001) differences between the four groups, i.e., high and low *cyp19a1a* expressors of the LT and HT treatments. **b** Sex ratios of juvenile European sea bass sampled at 332 days post hatch. Stacked bars showing male (blue) and female (red) percent in the low (LT) and high (HT) temperature groups. Statistical differences (*P* < 0.001) between groups are marked with three asterisks. Sample size: LT, 66 fish; HT, 85 fish

At 170 dph, from each temperature group we randomly sampled 20 fish (total 40 fish). However, since, as stated above, elevated temperature results in masculinization of some females, within the randomly selected fish we choose for microarray analysis, in each group, 5 fish with high *cyp19a1a* levels, which are typically associated with female development, and for this we used a previously validated clustering method based on *cyp19a1a* qRT-PCR expression levels (Fig. 1a). One-way ANOVA showed statistical differences due to the expression levels, temperature treatment and their interaction (*P* < 0.001). Thus, in the LT or control group, fish with high *cyp19a1a* levels undoubtedly represented developing females, whereas in the HT group fish with high *cyp19a1a* levels (but lower than the ones in the control group) also represented females that had been subjected to elevated temperature. It is important to state that despite overall differences in the sampled fish (Table 1) there were no differences in SL or BW between the 5 fish selected from the two groups for microarray analysis. Thus, the observed transcriptomic differences between HT and LT fish (see below) were due to temperature and not to size-related differences in gonadal development.

Visual assessment of the sex ratio of 332 dph juveniles combined with histological verification showed that the LT group had 40.0 % males while the HT group had 77.8 % males (*P* < 0.001) (Fig. 1b), showing a masculinizing effect of the elevated temperature. However, within each sex no differences were observed in the presence and in the abundance of the different cellular types between the two temperature groups. Females had immature ovaries containing oocytes at the cortical alveolar (CA) stage while males had testis containing all germ cell types, including spermatozoa (Additional file 2: Figure S1).

The GSI percent values at 332 dph for LT males and females were 0.095 ± 0.0004 and 0.111 ± 0.0002, respectively, and for HT males and females were 0.090 ± 0.0002 and 0.161 ± 0.0003, respectively. A two-way ANOVA analysis showed statistical differences due to sex (*P* = 0.032) but no effect due to the thermal treatment or to the interaction between sex and temperature.

### Microarray analysis

**Fig. 2:**
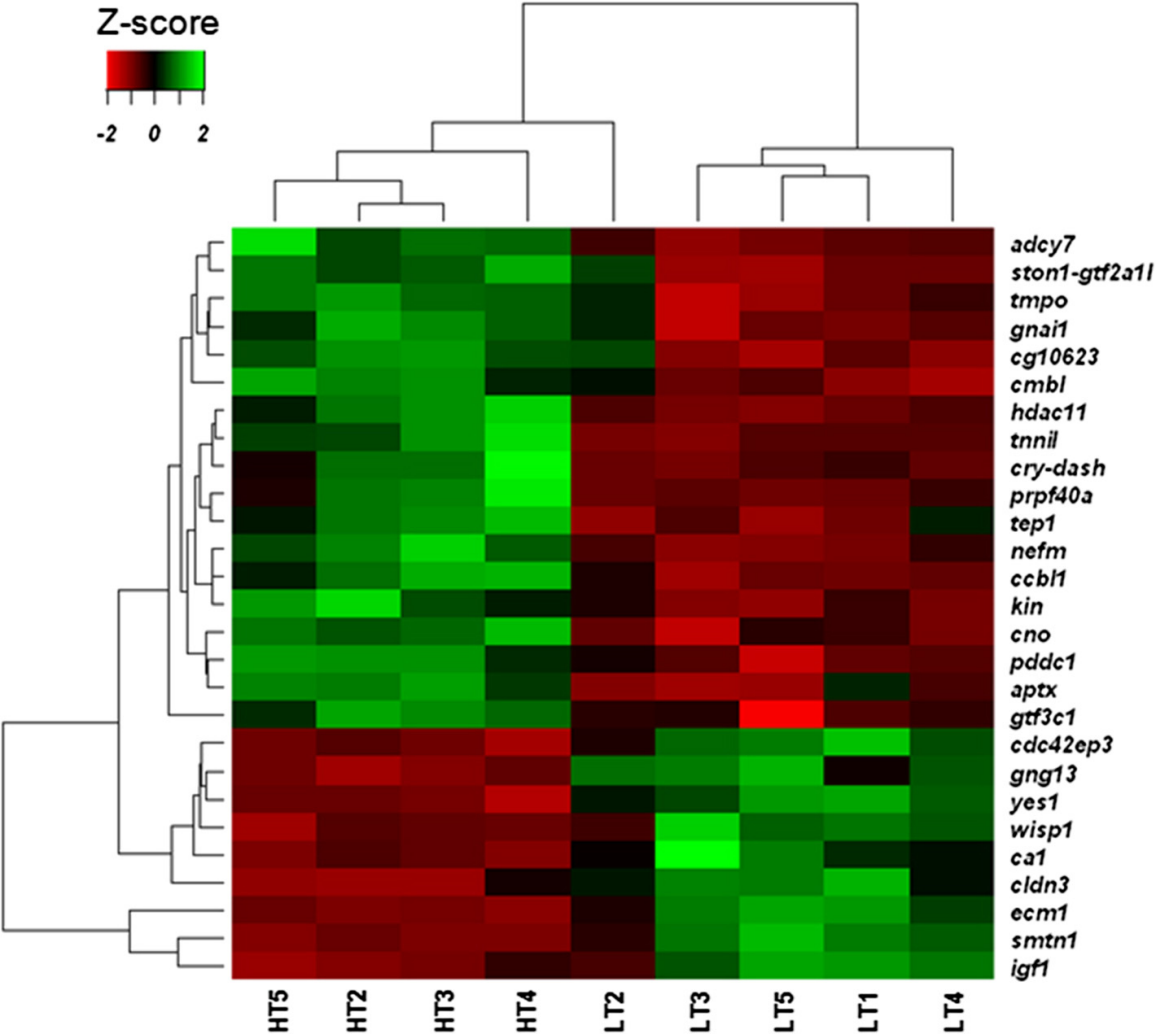
Heatmap of the microarray expression data for the 18 up- and 9 downregulated genes, where each row represents a gene and each column represents an individual fish (LT = 5 individuals and HT = 4 individuals). Key color representing the level of expression (green: high expression and red: low expression). The dendograms provide information of the similarity between genes and between the different samples. Notice that all HT samples and all but one LT samples cluster together. LT, low temperature group; HT, high temperature group. See Additional file 1: Table S2 for a complete list of gene names and abbreviations

Microarray analysis of sexually differentiating gonads at 170 dph obtained from fish with a high *cyp19a1a* expression, i.e., putative females, revealed the presence of 27 significantly and differentially expressed (DE) genes when comparing the HT vs. the LT group (Additional file 1: Table S2), of which 18 genes were upregulated (18/1360 non repeated probes) and 9 were downregulated (9/4789 non repeated probes). A heatmap representation of the DE genes grouped fish according to their thermal history, with the exception of one LT fish, which had an intermediate position, and one HT fish, which was classified as an outlier by a Principal Component Analysis and was not further considered in the analysis (Fig. 2). Some of the upregulated genes were related to reproduction, i.e., cryptochrome DASH (*cry-dash*) and troponin I (*tnnI*), or to epigenetic gene expression regulation, i.e., histone deacetylase 11 (*hdac11*). Some of the downregulated genes also showed reproduction-related functions such as cdc42 effector protein 3 (*cdc42ep3*), insulin-like growth factor (*igf1*) or smoothelin (*smtn1*) (see Additional file 1: Table S3 for a complete list of DE genes and their functions).

The AMIGO web-based tool was used to recover the sequence of these DE genes and those sequences were then uploaded to Blast2GO in order to enrich results with GO terms and extract more information about these DE genes. A Fisher’s exact test with multiple testing corrections for False Discovery Rate (FDR) showed that five GO term categories were overrepresented when compared to a reference test containing all the annotated sequences from our custom-made array (Additional file 1: Table S4).

Further analysis of the GO terms provided their distribution among the three main categories: biological process, molecular function and cell component for the up- and downregulated genes separately (Additional file 2: Figures S2 and S3, respectively). The Kyoto Encyclopedia of Genes and Genomes (KEGG) database provided more information on the pathways containing these DE genes (Additional file 1: Table S5). Several pathways involved in protein synthesis as well as in immunological processes were found.

### Microarray validation

**Fig. 3:**
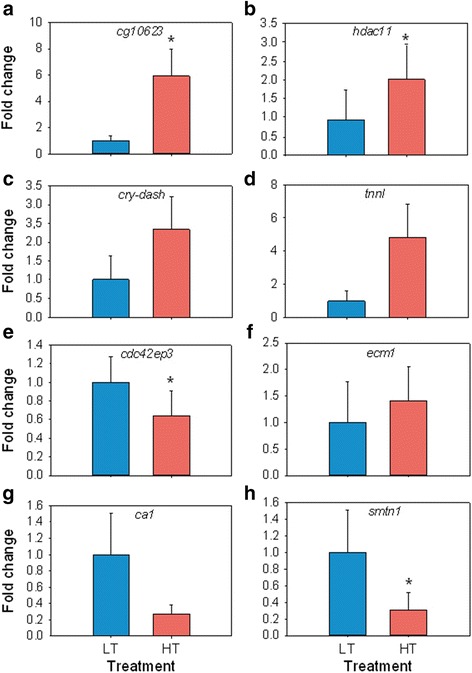
Validation of microarray results by analyzing ten fish by qRT-PCR according to treatment (LT, low temperature group; HT, high temperature group). **a**-**d** Four upregulated genes in the HT vs. the LT group comparison: DmeI_CG10623 (*cg10623*); histone deacetylase 11 (*hdac11*); cryptochrome DASH (*cry-dash*) and troponin I (*tnnI*). **e**-**h** Four downregulated genes for the same comparison: cell division cycle 42 effector protein 3 (*cdc42ep3*); extracellular matrix protein 1 (*ecm1*); carbonic anhydrase 1 (*ca1*) and smoothelin (*smtn1*). Data as mean ± SEM. Letters mark statistical significance (*P* < 0.05) between groups. Ten fish (5 fish per group) were analyzed by a qRT-PCR

Out of the 27 DE genes, we selected eight (4 up- and 4 downregulated genes), i.e., about one third of the DE genes, for validation by qRT-PCR. These genes were selected because they are known to be involved in reproduction in other species or because they are involved in epigenetic regulatory mechanisms. In addition, together they exhibited a wide dynamic range of changes. qRT-PCR analysis showed significant differences (*P* < 0.05) for *cg10623* and *hdac11* among the upregulated genes and for the *cdc42ep3* and *smtn1* among the downregulated ones (Fig. 3). Importantly, all but one (*ecm1*) of these selected genes had the same direction of change (up- or downregulation) when analyzed by qRT-PCR as compared to microarray results. We consider this sufficient for the validation of the microarray.

### Enrichment analysis

**Fig. 4:**
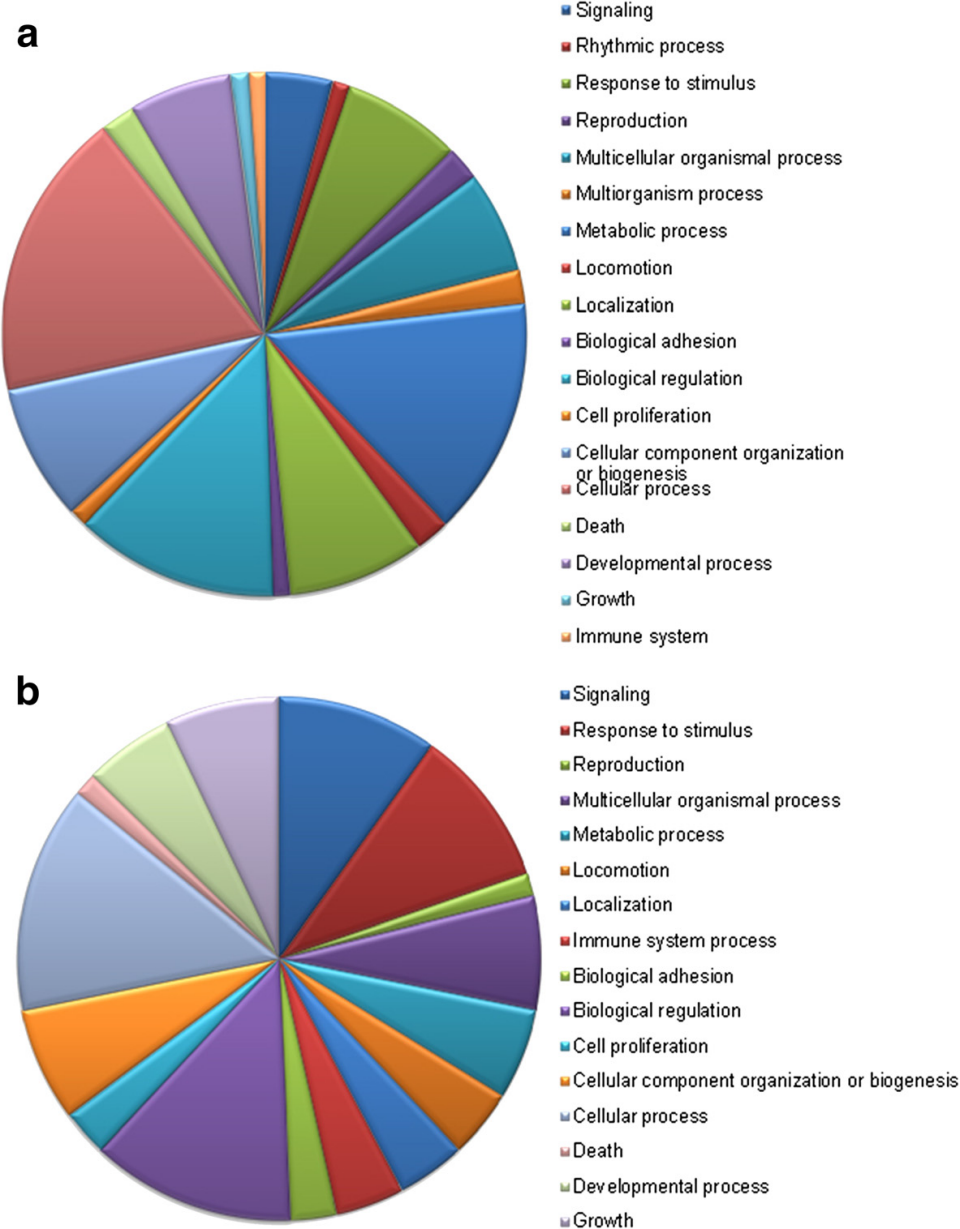
Biological process-related GO terms for the HT versus LT group comparison. **a** Biological process GO terms for the upregulated genes, and (**b**) for the downregulated genes

A GO enrichment analysis of the DE genes showed up- and downregulation of the same BP categories, albeit containing different DE genes (Fig. 4a and 4b). These altered categories contained more GO terms that were upregulated and mainly related to metabolic and cellular processes (14.43 % and 17.53 %, respectively), while other processes such as reproduction (1.41 %), growth (7.04 %), immune processes (4.23 %) or signaling (9.86 %) were downregulated at elevated temperatures. Regarding MF GO categories (Additional file 2: Figure S2A and S3A), the catalytic and binding activities were the most represented subcategories for both up- and downregulated GO terms. Analysis of the CC categories showed that upregulated processes were taking place mainly in the organelle (14/57 GOs), macromolecular complex (9/57 GOs), membrane-enclosed lumen (8/57 GOs) or membrane (7/57 GOs) (Additional file 2: Figure S2B), while the downregulated processes were taking place in the membrane, organelle, macromolecular complex (6/36 GOs) or extracellular region (5/36 GOs) (Additional file 2: Figure S3B). Further analysis of the GO enriched terms of the DE genes in comparison to the microarray reference set (Additional file 1: Table S4), showed that terms related to the negative regulation of the nerve impulse and synaptic transmission were overrepresented, as well as the adenylate cyclase inhibiting G-protein coupled receptor signaling pathway (implying a decrease in cAMP concentration).

Blast2GO analysis of the DE genes showed that eleven of the 13 differentially regulated pathways (Additional file 1: Table S5) had higher expression in HT fish than in LT fish and that these pathways were related to catabolism (amino acid metabolism), biosynthesis (tropane, piperidine and pyridine alkaloid) or signal transduction (phosphatidylinositol signaling system). However, the downregulated pathways were related, in agreement with the results of the GO term analysis, to immunology (T-cell receptor signaling pathway) and Nitrogen metabolism. DAVID analysis of the DE genes showed that there were 23 upregulated categories, of which four were highly significant: 1) progesterone-mediated oocyte maturation (*gnai1, adcy7* and *igf1*), 2) tight junction (*gnai1, cldn3* and *yes1*), 3) chemokine signaling (*gnai1, adcy7* and *gng13*), and 4) hormone-mediated signaling (*adcy7* and *gng13*) pathways. In all of them, *adcy7* is involved as a signaling initiation factor. After running an annotation clustering with DAVID, seven clusters were downregulated and related to cell component, DNA binding, transcription and signaling processes.

### Reproduction and stress-related genes

**Fig. 5:**
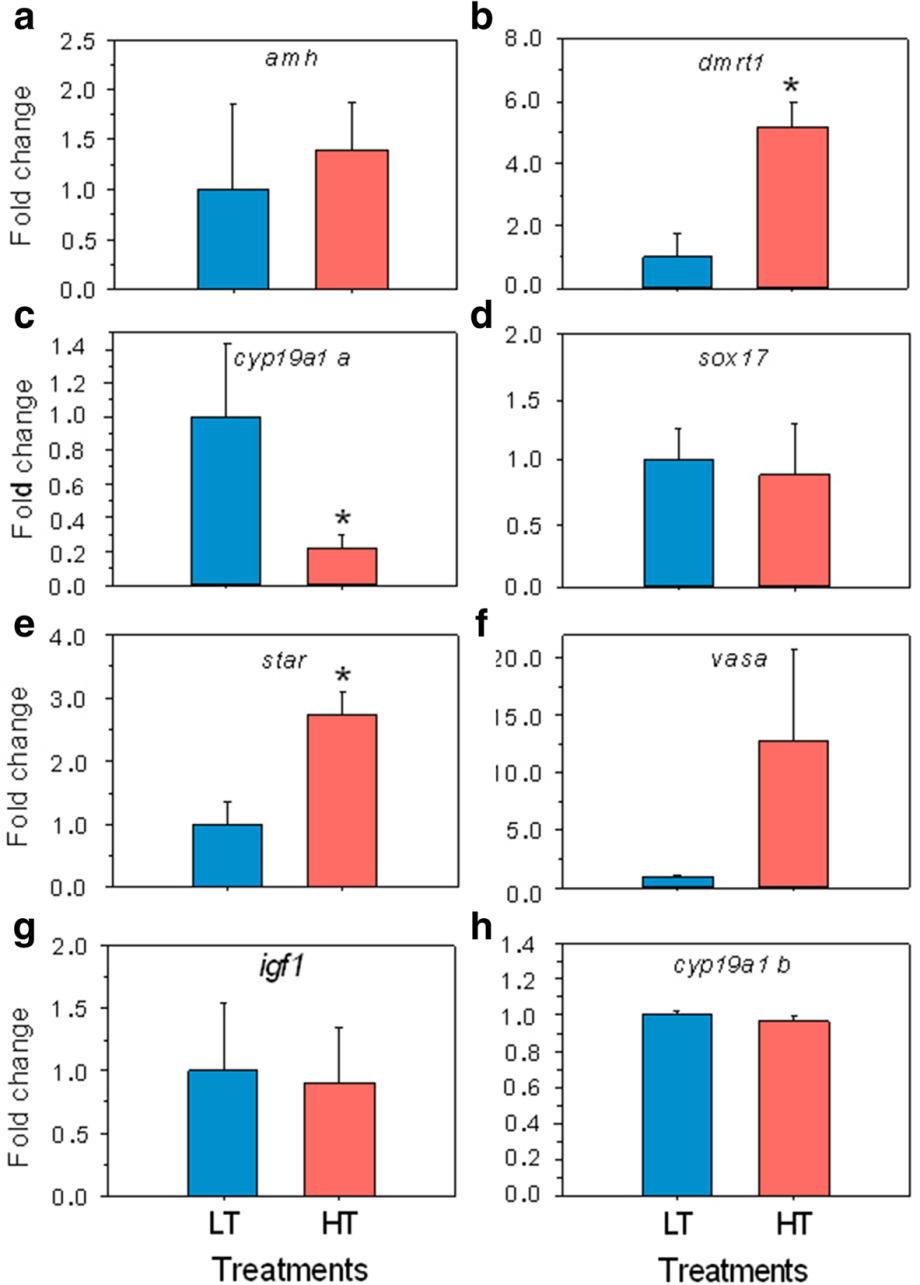
Quantitative RT-PCR results for eight known sex differentiation-related genes per temperature treatment groups. **a**-**d** Male pathway: anti-Müllerian hormone (*amh*), doublesex- and mab-3-related transcription factor 1 (*dmrt1*); female pathway: cytochrome P450, family19, subfamily A, polypeptide 1a (*cyp19a1a*) and SRY-related HMG-box transcription factor SOX17 (*sox17*), respectively. **e** steroidogenic acute regulatory protein (*star*), (**f**) vasa protein (*vasa*), (**g**) insulin-like growth factor 1 (*igf1*) and (**h**) cytochrome P450, family19, subfamily A, polypeptide 1b (*cyp19a1b*). Data as mean ± SEM. Asterisk marks statistical differences between groups (*P* < 0.05). Ten fish (5 fish per group) were analyzed by a qRT-PCR

**Fig. 6:**
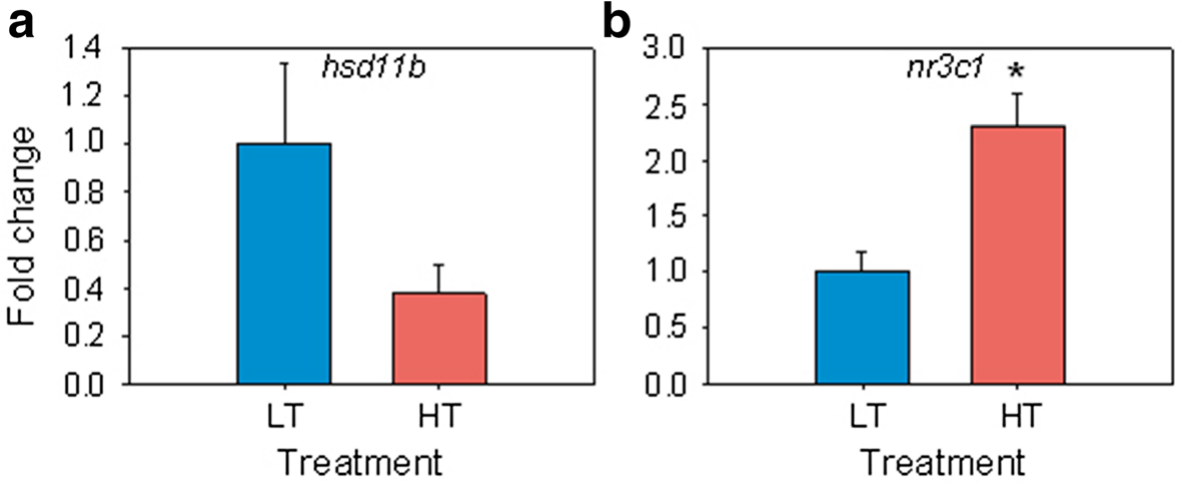
Quantitative RT-PCR results for (**a**) 11β-hydroxysteroid dehydrogenase (*hsd11b1*) and (**b**) glucocorticoid receptor (*nr3c1*). Data as mean ± SEM. Asterisk marks statistical differences between groups (*P* < 0.05). Ten fish (5 fish per group) were analyzed by a qRT-PCR

Fifteen known reproduction-related genes were analyzed by qRT-PCR (Additional file 1: Table S6). Results showed that genes involved in testicular differentiation, such as doublesex-mab-3-related transcription factor 1 (*dmrt1*), were significantly (*P* < 0.05) upregulated in HT fish, as also was the steroidogenic acute regulatory protein (*star*). In contrast, some genes involved in ovarian differentiation, such as aromatase (*cyp19a1a*), were significantly (*P* < 0.05) downregulated in HT fish, as also was aquaporin 1 (*aq1*). The expression of eight of these genes is shown in Fig. 5. Since activation of the stress response has been associated with temperature effects on sex ratios in fish [50–53], we tested the effects of HT in our model in two stress response-related genes; the 11ß-hydroxysteroid dehydrogenase (*hsd11b*) and the glucocorticoid receptor (*nr3c1*), involved in cortisol synthesis and response. While *nr3c1* was significantly (*P* < 0.05) upregulated in HT fish, *hsd11b* was not significantly different (Fig. 6).

### Epigenetic mechanisms-related genes

**Fig. 7:**
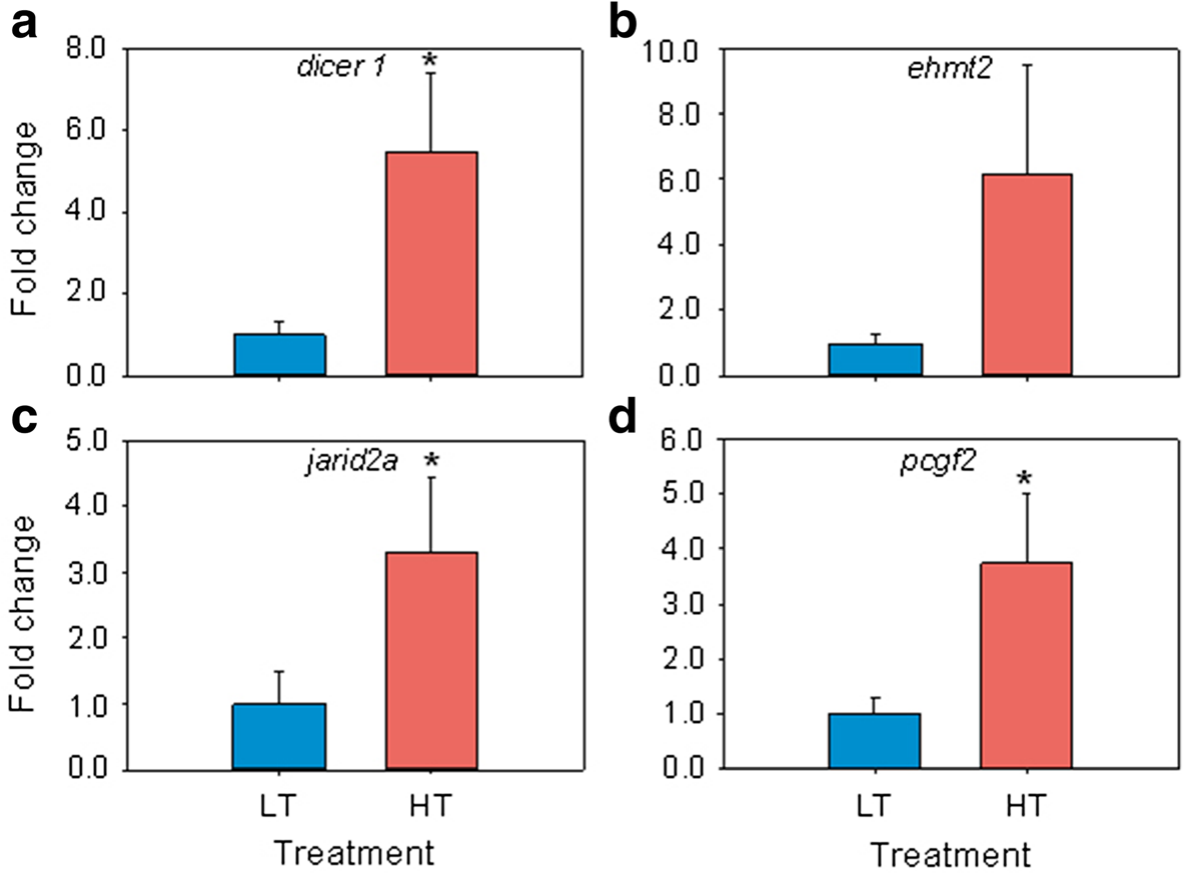
Quantitative RT-PCR results for the epigenetic regulatory mechanisms-related genes. (**a**-**d**) endoribonuclease Dicer (*dicer1*), euchromatic histone-lysine N-methyltransferase 2 (*ehmt2*), protein Jumonji (*jarid2a*) and polycomb group ring finger 2 (*pcgf2*). Data as mean ± SEM. Asterisks mark statistical differences between groups (*P* < 0.05). Ten fish (5 fish per group) were analyzed by a qRT-PCR

According to a growing body of evidence, several genes involved in epigenetic regulatory mechanisms have been implicated in sex determination/differentiation ([54], for a review). Based on this, we analyzed the genes related to epigenetic mechanisms and implicated in sex determination/differentiation [54] that were present in our microarray (Additional file 1: Table S7) even if they were found to be not DE. Seven genes, representative of different categories of epigenetic regulatory mechanisms, including *dicer 1*, a helicase needed to produce an active small RNA component that represses gene expression; *ehmt2*, a histone methyltransferase; *jarid2a*, a DNA-binding protein that acts as a transcriptional repressor; *pcgf2*, which contains a RING finger motif and forms protein-protein interactions to maintain transcriptional repression; *hdac11*, a histone deacetylase; *mettl22*, a methyltransferase-like protein; and *suz12*, a suppressor of trithorax zeste 12 homolog gene, were selected and analyzed by qRT-PCR. Four of these genes were upregulated (*P* <0.05) in the HT group: *dicer1, jarid2a, pcgf2* and *hdac11*) (Fig. 7).

## Discussion

To the best of our knowledge, this is the first study on the analysis of the gonadal transcriptome at the time of sex differentiation, using a species-specific microarray, of fish previously exposed to elevated temperature during the thermosensitive period. Since we took care that the 5 fish selected from each group for microarray analysis at 170 dph (high *cyp19a1a* expressors) had the same size, it is safe to assume that differences in gene expression observed are solely due to temperature, not to differences in growth.

Consistent with previous studies [46, 49], temperature induced a male-biased sex ratio (~80 % males). The higher GSI values for HT-treated females observed at one year of age may be due to a persistent effect on gonadal growth relative to somatic growth with no obvious effect at the histological level.

The number of DE genes was low as could be anticipated because in the HT vs. LT comparison we actually compared fish of the same age, at a similar developmental stage and similar size, as just stated above, and most likely of the same sex, females, as assessed by high *cyp19a1a* expression levels, a good marker of phenotypic sex. This is actually what we intended since females, not males, are the ones mainly affected by temperature. Moreover, at 170 dph gonads were cleanly isolated and there was no contamination of the surrounding tissues. What we could gain by looking at earlier age, e.g., at the end of the TSP at about 60 dph, we would lose by having to include non-gonadal tissues (since it is impossible to dissect the gonads apart at earlier ages).

Among the genes analyzed by qRT-PCR, there were three patterns of response to heat (Figs. 3, 5, 6, and Additional file 1: Table S6). Many genes had increased levels of expression in the HT group. Among them, there were genes upregulated during normal testis differentiation such as *dmrt1*, in accordance with the masculinizing effect of elevated temperatures [46, 49].. The vasa helicase is a germ cell marker known to be more expressed in ovaries than in testis and previously found to be upregulated by elevated temperatures [55]. In our study the difference in vasa expression levels did not reach statistical significance. Cholesterol is important in maintaining membrane integrity and sterol synthesis and its levels have previously been found also to be increased by temperature [2]. However, genes ultimately related to steroid hormone production were not significantly affected in the present study, including the gene encoding gonadotropin-releasing hormone (gnrh) [56, 57], and star, which is involved in cholesterol import [58]. In our study, genes such as *wisp1*, known to be involved in cellular growth, were downregulated by heat, whereas genes such as *nr3c1*, related to immune system regulation responses, were upregulated, suggesting growth and immunological stress response adjustments in the gonads.

There were other downregulated genes. Among these there was *cyp19a1a*, confirming results of an independent experiment aimed to find molecular signatures of male and female differentiation [17]. Aquaporin 1 (*aqp1*), a water channel protein which plays a major role in oocyte hydration in fish [59], was also downregulated by heat. The steroidogenic enzyme 11ß-hydroxysteroid-dehydrogenase 1 (*hsd11b*), which converts the stress hormone cortisol into the inactive metabolite cortisone, and also converts 11ß-hydroxy androgens such as 11ß-hydroxyadrostenedione into 11-ketotestosterone, a potent piscine androgen [60], was not significantly affected by heat. In contrast, the glucocorticoid receptor (*nr3c1*) was upregulated in the HT group with significant differences with respect to the LT group. These results are interesting because in the pejerrey (*Odontesthes bonaeriensis*), a fish with TSD, Fernandino *et al*. [53] observed upregulation of both *hsd11* and *nr3c1* in masculinized gonads by HT and attributed this to the fact that HT elicits both masculinizing and stress responses. In our study, *nr3c1* was upregulated 110 days after the end of the temperature treatment, suggesting a persistent stress response probably maintained by an epigenetic regulatory mechanism. In fact, studies in rodents have shown that *nr3c1* is able to exhibit sustained expression, even a long time after the stimulus ended, through changes in methylation of its regulatory region [61, 62]. The differences between our results and those of Fernandino *et al*. [53] with the pejerrey suggests that gender or developmental stage may be important in explaining these differences, since while Fernandino et al. [53] sampled juvenile, sexually differentiated males, in our case we selected not only sexually differentiating fish but also those ones that exhibited *cyp9a1a* expression levels at 170 dph compatible with ovarian differentiation. Furthermore, when comparing our results with those obtained with microarray analysis of dimorphic gene expression in a turtle with TSD [63], genes that are normally highly expressed in the testis such as *dmrt1* were also upregulated in our study while genes such as *cyp19a1a* were downregulated due to temperature, highlighting the conserved masculinizing effect of heat across different vertebrate groups.

The third pattern of response was represented by genes whose expression was not affected by heat, including *sox17*, a gene that has been related with ovarian development in the European sea bass and other fish [64]. The brain aromatase gene (*cyp19a1b*) belonged to this group, thus corroborating earlier observations of our group [49], and opposite to the increase observed in tilapia when applying HT during early development [65]. Other genes also in this group were *tesc*, important during male gonadal development, and *col18a1*, implicated in organ morphogenesis.

Since the hypermethylation of the *cyp19a1a* promoter in both females and masculinized females at one year of age was not so evident at 170 dph [49], it may be that other epigenetic regulatory mechanisms are responsible for the “memory” of early HT exposure. To gain further evidence, we examined the expression of seven epigenetic regulatory mechanisms-related genes that have been directly or indirectly connected with sex determination and gonadogenesis [54] and were present in our microarray (Additional file 1: Table S7). The selected genes for qRT-PCR analysis were *hdac11, jarid2a, ehmt2, dicer1, suz12, pcgf2* and *mettl22* to have representatives of different epigenetic mechanisms. Interestingly, four of them were upregulated in the HT group, displaying significant differences (*P* < 0.05): *hdac11, jarid2a, dicer1* and *pcgf2*. Although further studies are clearly needed, it is interesting to note that, although in different ways, these genes are involved in transcriptional repression functions, which here may be connected with the long-lasting effects of early heat exposure.

Whole gonad transcriptomic analysis showed 27 DE genes (18 up- and 9 downregulated; Fig. 2 and Additional file 1: Table S3). Some of these DE genes were related to metabolic processes, and some to epigenetic regulatory mechanism including *cg10623* (methyltransferase), *hdac11* (histone deacetylase) or *tep1* (related to DNA methylation increase). Other genes are involved in reproductive processes in other species such as *cdc42ep3*, where the mRNA interacts with the human fertility protein PUMILIO2 in the testis [66]. *cry-dash*, has an ancestral circadian role in light perception and related to massive spawning in corals during full moon [67] while *smtn1* is a regulator of the progesterone receptor during mice pregnancy [68] and *tnn1* controls ovulatory contraction of non-striated actomyosin networks in *Caenorhabditis elegans* [69].

Enrichment analysis showed an upregulation of the overall catalytic activity, a process known to be affected by heat since elevated temperature produces changes in chemical reaction rates and increases protein denaturalization [3]. This corresponded to an overrepresentation of the catalytic pathways and of the signal transduction due to heat. The observation that most of the altered pathways were related to catabolism and signal reception-transmission corroborates the idea that the effects of heat were still persistent 110 days after the thermal treatment had finished.

In order to further understand the biological meaning of the set of DE genes, DAVID clustering analysis showed that DNA binding and transcription were enriched, suggesting that despite protein and amino acid catabolism, protein synthesis and replacement was also occurring in HT gonads, most likely to compensate for the destabilizing temperature effects on protein structure. Likewise, downregulation of the immunology-related pathway may be because our samples were obtained from differentiating females (progesterone-mediated oocyte maturation is affected, as also is the hormone-mediated signaling).

Cossins *et al.* [32] investigated the transcriptomal response of seven carp tissues to cold. From that study, we selected the 15 genes that showed the greatest increase in expression in response to cold: 92 kDa type IV collagenase precursor (gelatinase), ADP/ATP translocase 1, ATP-binding cassette, subfamily F member 2, apolipoproteins, RNA-binding protein, NADP-dependent malic enzyme, mitochondrial uncoupling protein 3, calmodulin, cofilin, granulin alpha, tubulins alpha, beta and gamma chains and high mobility group 1. All are involved in a variety of functions including protein turnover, unsaturated fatty acid synthesis, homeostasis or stress protein production. Most of these 15 genes were present in our microarray and, interestingly, when we examined their behavior, we found that, in general, expression levels tended to be lower in our HT group, although without significant differences (Additional file 1: Table S8). Thus, many of the genes that Cossins *et al*. [32] found upregulated by cold in the carp intestine with transport and regulatory functions (see Additional file 1: Table S8) had, when present, a downward tendency in the HT gonads. We realize that this may be an anecdotal coincidence, but the observation that the above-mentioned genes seem to behave depending on whether fish are exposed to heat or cold and regardless of tissue or species, warrants their further study in other species exposed to temperature changes. It could be possible that these genes could serve as markers of previous thermal history.

In contrast to what has been observed in killifish livers [30], heat shock proteins were downregulated in our study, suggesting that the short heat exposure took place enough time ago to allow the return to their normal expression levels and become downregulated. In addition, cholesterol and genes involved in the lipid metabolism were affected by heat. Thus, for example, cholesterol synthase and HMG-CoA reductase were downregulated as a result of chronic temperature elevation, as previously reported by Podrabsky and Somero [30]. The presence of translation elongation factors or proteasomes with a high level of expression due to heat is also in agreement with previous studies in other tissues [29, 30].

Apart from common transcriptional responses to heat, each tissue seems to have different strategies to cope with temperature changes: brain modulates glycolytic activity, liver turns on lipid metabolism and muscle remodels its contractile apparatus [29]. From the present study, we can add that gonads increase catabolism and signal transduction, but reproductive and immune related functions decrease. This is in agreement with the documented deleterious effects of high temperature on gonadal function [70].

## Conclusion

This paper provides information on the effect of temperature at the whole gonadal transcriptomic level and at the time of sex differentiation in a fish with mixed genetic and environmental sex determination. At about 3–4 months after the end of exposure, downregulation of the expression of female-related genes such as *cyp19a1a* and an increase in male-related ones such as *dmrt1* was readily observed. Furthermore, some signaling, catabolic, biosynthetic, growth and reproduction pathways were still affected. Our study shows that the response to a change in the early environment, in this case of temperature, is evident months after the environmental perturbation has finished. These transcriptomic changes determine the course of sex differentiation and ultimately alter the population sex ratio. Furthermore, this study, in addition to reporting changes in the expression of cognate genes and signaling pathways related to vertebrate sex differentiation, identifies genes previously not implicated in thermal-induced gonadal development, which seem conserved across species, as well as genes related to epigenetic regulatory mechanisms responsible for acquiring and maintaining cell identity, of which some were upregulated by high temperatures. This data provide an advance to our understanding of the underlying mechanisms responsible for the environmentally driven transcriptomic changes, leading, in turn, to changes in an important phenotype such as the sexual phenotype.

## Methods

### Animals and rearing conditions

One-day-post hatch (dph) European sea bass larvae were obtained from a commercial hatchery and were transported to our facilities in PVC transport bags filled with oxygen and seawater. Rearing conditions and handling methods were as previously described [71], except for the temperature treatment (see below).

Fish were treated in agreement with the European Convention for the Protection of Animals used for Experimental and Scientific Purposes (ETS Nu 123, 01/01/91). Our facilities are approved for animal experimentation by the Ministry of Agriculture and Fisheries (certificate number 08039-46-A) in accordance with Spanish law (Real Decreto 223 of March 1988) and the experimental protocol was approved by the Spanish National Research Council (CSIC) Ethics Committee within project AGL2010-15939). Animals were sacrificed by an overdose of 2-phenoxyethanol followed by severing of the spinal cord.

### Experimental design

Larvae were divided in two 650-l tanks and maintained at 17 °C, a temperature known to avoid temperature effects on sex ratio [46], for the first 20 dph. Then, in one tank the temperature was increased to 21 °C (high temperature, HT group), while in the other it was decreased to 15 °C (low temperature, LT group). In both cases, temperature was modified at a ratio of 0.5 °C/day. At 60 dph, temperature in the LT group was stepwise increased in order to match the temperature of the HT group. Then, at ~220 dph, temperature of both groups was left to follow the natural fluctuations. Thus, the only difference between the LT and the HT groups in terms of rearing conditions was in the temperature experienced during the 20–60 dph period (Additional file 2: Figure S4).

### Samplings

Periodic samplings were carried out, where length (SL; precision 1 mm) and body weight (BW; precision 0.01 g) were assessed for all fish in each group by anesthetizing them with adjusted doses of 2-phenoxyethanol (2PE; 0.2 ml · l^−1^). At 170 dph, coinciding with the period of histological sex differentiation [72], and at 332 dph, when gonadal sex is firmly established, a sample of fish (*n* = 40 at 170 dph; *n* = 151 at 332 dph) were randomly taken from each group and sacrificed with an overdose of 2PE. In the European sea bass, the first differences in *cyp19a1a* start to become apparent at 120 dph [17] but histological differentiation does not start to be evident until 150 dph. Earlier sampling could perhaps target the early transcriptomic events associated with temperature sex-reversal mechanisms. However, the primary goal of this study was to target gonads during sex differentiation in order to understand the underlying process at that time in presumptive females of the HT group when compared to the LT group. In addition, with younger fish the dissected gonads can be contaminated with surrounding tissue. In this regard, analyzing gene expression changes due to temperature at the end of the TSP at about 60 dph or even in fish two months older than that would be quite difficult because at this age European sea bass gonads are so small that it is almost impossible to dissect them without taking part of the surrounding tissue, which could alter gene expression data.

To minimize external differences not related to the thermal treatment, the microarray comparison was done between fish of the same age, at a similar developmental stage (no obvious differences in size were observed) and most likely of the same sex, females, as assessed by *cyp19a1a* expression levels, a good marker of phenotypic sex and also of temperature disruption. Thus, we compared normally developing females (LT group) with females previously exposed to elevated temperature (group HT).

At 170 dph, sexually differentiating gonads were cleanly dissected out without any contamination of the surrounding tissues and snap-frozen in liquid Nitrogen for transcriptomic analysis. At 332 dph, gonads were dissected out and weighted (precision 0.01 g) to calculate the gonadosomatic index (GSI) as previously described [46, 71]. Gonads were fixed in 4 % paraformaldehyde (PF) in PBS. Sex ratio of the population was visually assessed (*n* = 151 fish total; HT: *n* = 85 and LT: *n* = 66 fish) coinciding with the last sampling at 332 dph.

Twenty PF-fixed 332 dph gonads per group (10 of each sex) were used for sex assessment and to determine the stage of gonadal development after staining with hematoxylin-eosin (Additional file 2: Figure S1) following conventional histological procedures. Female and male developmental stages were assessed according to Brown-Peterson et al. [72]. Stages of oocyte maturation were classified as: cortical alveolar (CA), primary growth (PG) and primary vitellogenic stage (Vtg1). Male germ cells in different stages of spermatogenesis were classified as: primary spermatogonia (SpgA), primary spermatocytes (Scp1), secondary spermatocytes (Spc2), spermatids (Spd) and spermatozoa (Spz).

### RNA extraction and cDNA synthesis

Total RNA was purified from 170 dph isolated juvenile sexually differentiating gonads with Trizol reagent (Invitrogen- Live Technologies, Scotland, UK). The quality and concentration of the RNA were assessed with a ND-1000 spectrophotometer (NanoDrop Technologies) based on A260 absorbance and checked on a 1 % agarose/formaldehyde gel.

Two hundred nanograms of total RNA were used for cDNA synthesis using SuperScript III Reverse Transcriptase (Invitrogen, Spain) and random hexamer primers (Invitrogen, Spain) following the manufacturer’s instructions and were then treated with E.coli RNAse H in order to remove complementary RNA.

### Quantitative real time PCR (qRT-PCR)

Real time PCRs were performed with two purposes. First, to select 5 fish per treatment at 170 dph for further microarray analysis based on 2DCt *cyp19a1a* qRT-PCR values after a two-step clustering analysis (see statistical section below). At 170 dph, sex differentiation is taking place and differences in *cyp19a1a* expression are evident between presumptive future males and females starting after 120 dph [17]. Thus, determination of *cyp19a1a* levels allowed selecting fish with the highest *cyp19a1a* levels by increasing the chances to concentrate our efforts on the effects of temperature on presumptive future females, which are masculinized in response to temperature. Second, to validate microarray results (*n* = 5 individuals/treatment) and analyze genes that are important either for sex differentiation or related to epigenetic regulatory mechanisms (see Additional file 1: Table S9 for a gene glossary). cDNA was diluted 1:10 for the amplification of the target genes and 1:500 for the housekeeping, reference gene r18S. Primers were designed using Primer 3 Plus (http://www.bioinformatics.nl/cgi-bin/primer3plus/primer3plus.cgi/) (Additional file 1: Table S10). A melting curve analysis (95° for 15 s, 60° for 15 s and 95° for 15 s) was performed after the amplification phase to analyze primer specificity (Additional file 1: Table S10). Real-time PCR was performed on an ABI 7900HT (Applied Biosystems) with the following program: an initial UDG decontamination cycle at 50° for 2 min, followed by an activation step of 10 min at 95° and then 40 cycles of 15 s denaturation at 95° and a 1 min annealing/extension step at 60°. Finally, a dissociation step of 15 s at 95° followed by 15 s at 60° was added.

Ten samples per group were run in triplicate in 384-well plates in a final volume of 10 μl per well. Each well contained a mix of 5 μl of SYBRGreen Supermix (Applied Biosystems), 2 μl distilled water, 2 μl primer mix (forward and reverse primers at 10 μM concentration) and 1 μl of cDNA. Controls lacking either cDNA or primers were included per duplicate. Data was collected using SDS 2.3 software (Applied Biosystems) and gene expression levels were calculated using RQ Manager 1.2 (Applied Biosystems). Endogenous control gene r18S was used in all runs to calculate intra- and inter-assay variations. Ct values were adjusted for differences in efficiency of each primer set when analyzing the results, and expression of target genes was normalized to the reference gene (r18S) based on the Schmittgen and Livak [73] method.

### Microarray analysis

Microarray experiments consisted on the comparison of 5 individuals of each temperature group (LT and HT) sampled during the process of sex differentiation at 170 dph. Before microarray hybridizations, the integrity of the total RNA was verified in a 1 μl-sample with a Bioanalyzer 2100 fitted with the RNA 6000 Nano LabChip kit (Agilent, Spain) to assure consistency across samples. Only RNA samples of 100–200 ng/μl and RINs > 7 were used for microarray hybridizations. RNA labelling, hybridizations, and scanning were performed according to the manufacturer’s instructions. Briefly, total RNA (100 ng) was amplified and Cy3-labeled with Agilent’s One-Color Microarray-Based Gene Expression Analysis (Low Input Quick Amp Labelling kit), along with Agilent’s One-Color RNA SpikeIn Kit. After labelling, cRNA was purified with RNeasy mini spin columns (Qiagen), quantified with the Nanodrop ND-1000 and verified using the Bioanalyzer 2100. Each sample (1.65 μg) was hybridized to a custom-made European sea bass microarray containing a total of 17,917 probes (Agilent ID 023790) at 65° for 17 h using Agilent’s GE Hybridization Kit. Washes were conducted as recommended by the manufacturer using Agilent’s Gene Expression Wash Pack with stabilization and drying solution. Arrays were scanned with Agilent Technologies Scanner, model G2505B. Spot intensities and other quality control features were extracted with Agilent’s Feature Extraction software version 10.4.0.0. The complete design has been submitted to Gene Expression Omnibus (GEO)-NCBI database (GSE52307) as well as the platform that validates the microarray (GPL13443).

### Statistical analysis of data

Prior to statistical analysis, the normality of data was checked with the Kolmogorov-Smirnov’s test and the homoscedasticity of variance with the Levene’s test. Data of continuous variables was log transformed when needed. Percentage data such as GSI were arcsine transformed. One-way analysis of variance (ANOVA) was performed to check statistical differences between temperature treatments for SL, BW and GSI data sets including *cyp19a1a* expression levels when considering sex and thermal treatment as separate groups (see *qRT-PCR* section below). *Post hoc* multiple comparisons were carried out using the Tukey’s HSD test. The Student’s *t*-test was used to pairwise compare high *vs*. low aromatase expressors between thermal treatments. The Chi-square test with Yates correction was used to analyze differences in sex ratios. Differences were accepted as significant when *P* < 0.05. Unless otherwise stated, statistical analyses were performed using IBM SPSS Statistics v19.

Quantitative RT-PCR statistical analysis was performed using 2DCt from the processed data [73]. 2DCt results were then checked for normality, homoscedasticity of variance and the Student’s t- test was used to assess differences between treatments.

A two-step cluster analysis using 2DCt *cyp19a1a* qRT-PCR values was used to differentiate among high and low *cyp19a1a* expressors in both the LT and HT groups at 170 dph as previously described [17]. These analyses were performed using PAST software [74].

Microarray raw data was taken from the Feature Extraction output files and was corrected for background noise using the normexp [75] method. To assure comparability across samples, quantile normalization [76] was used. A probe or replicate was considered reliable if its raw foreground intensity was at least two times higher than the respective background intensity and if it was neither saturated nor flagged by the Feature Extraction software. On our custom array design, most probes (64.7 %) were represented in two (or in some cases more) identical replicates. Mean intensities of probe replicates were taken in order to yield only one expression value per probe. A probe was considered reliable if at least half of its replicates were individually reliable, as defined above.

Differential expression analysis was carried out on all non-control probes with an empirical Bayes approach on linear models (limma) [77]. Results were corrected for multiple testing according to the False Discovery Rate (FDR) method [78]. Genes were selected as differentially expressed if they had an adjusted *p*-value <0.05, an absolute fold change (FC) >1.2 and were reliable, as defined above, in all samples. All statistical analyses were performed with the Bioconductor project (http://www.bioconductor.org/) in the R statistical environment (http://cran.rproject.org/) [79].

For gene annotation enrichment analysis, gene names, gene synonyms and gene functions were addressed using mostly Genecards (http://www.genecards.org/) and Uniprot (http://www.uniprot.org/). The web-based tool AMIGO (http://amigo.geneontology.org/amigo) Gene Ontology [80] was used to retrieve the differentially expressed (DE) gene sequences. After obtaining the sequences, Blast2GO software [81] was used to enrich GO term annotation and to analyze the altered KEGG pathways (http://www.genome.jp/kegg/) that include those DE genes in order to extract a broader biological meaning. Using Blast2GO a reference set containing all the genes from the custom-made microarray was analyzed and used to check if the GO terms were enriched in a test group (DE genes set) when compared to it by a Fisher’s Exact Test with Multiple Testing Correction of FDR [59]. Also DAVID (https://david.ncifcrf.gov/) [82, 83] was used to further analyze and verify the pathways to which the DE genes belong.

### Availability of supporting data

The complete design can be accessible at the Gene Expression Omnibus (GEO)-NCBI database (GSE52307) as well as the platform that validates the microarray (GPL13443); http://www.ncbi.nlm.nih.gov/geo/query/acc.cgi?token=gfingckwblqhpwh&acc=GSE52307.

## Additional files

Additional file 1: Table S1. Summary of the studies on the effects of temperature on gene expression in fish. **Table S2.** Differentially expressed genes summary results. **Table S3**. Differentially expressed genes summary results. **Table S4.** Fisher’s exact test with multiple testing correction of FDR depicting the over-represented GO terms. **Table S5.** Summary of the KEGG pathways from the up- and downregulated gene list. **Table S6.** Microarray versus qRT-PCR fold change results for 15 selected reproduction-related genes. **Table S7.** Epigenetic regulatory mechanisms- related genes implicated in sex differentiation as discussed in Piferrer [54] that are present in the European sea bass custom-made microarray used in this study. **Table S8.** Comparison between the microarray results of this study and those reported in the literature on effects of temperature at the transcriptomic level (Gracey et al. [29]; Podrabsky and Somero [30]; Cossins et al. [32]; Vergauwen et al. [38]; Chojnowski and Braun [63]). **Table S9.** Gene abbreviation glossary. **Table S10.** Quantitative QRT-PCR primer characteristics. (DOCX 128 kb) Additional file 2: Figure S1. Photomicrographs of one-year-old European sea bass gonads. (A) LT females, (B) HT females, (C) LT males and (D) HT males. Scale bar = 50 μm. **Figure S2.** GO terms results and classification in two main categories of the upregulated genes in the HT group: A, molecular function (MF); and B, cell component (CC). **Figure S3.** GO terms results and classification in two main categories of the downregulated genes in the HT group: A, molecular function (MF); and B, cell component (CC). **Figure S4.** Diagram on European sea bass sex differentiation events, experimental design and sampling strategy. On a calibrated age scale, the bottom panel illustrates the main events related to gonadal sex differentiation. The middle panel depicts the low (LT) and high (HT) temperature periods, matching the thermosensitive period (TSP). The boxes indicate the sampling for transcriptomic analysis in relation to age and events of sex differentiation. The top panel highlights the two main samplings of the experiment and the type of the performed analyses. (DOCX 1693 kb)
