# Supplementary material for: Lasting effects of early exposure to temperature on the gonadal transcriptome at the time of sex differentiation in the European sea bass, a fish with mixed genetic and environmental sex determination
Source: BMC Genomics. 2015 Sep 4;16(1):679. doi: 10.1186/s12864-015-1862-0 (PMC4560065; doi:10.1186/s12864-015-1862-0)
Supplement: Additional file 1: Table S1. — Summary of the studies on the effects of temperature on gene expression in fish. Table S2. Differentially expressed genes summary results. Table S3. Differentially expressed genes summary results. Table S4. Fisher’s exact test with multiple testing correction of FDR depicting the over-represented GO terms. Table S5. Summary of the KEGG pathways from the up- and downregulated gene list. Table S6. Microarray versus qRT-PCR fold change results for 15 selected reproduction-related genes. Table S7. Epigenetic regulatory mechanisms- related genes implicated in sex differentiation as discussed in Piferrer [54] that are present in the European sea bass custom-made microarray used in this study. Table S8. Comparison between the microarray results of this study and those reported in the literature on effects of temperature at the transcriptomic level (Gracey et al. [29]; Podrabsky and Somero [30]; Cossins et al. [32]; Vergauwen et al. [38]; Chojnowski and Braun [63]). Table S9. Gene abbreviation glossary. Table S10. Quantitative QRT-PCR primer characteristics. (DOCX 128 kb) [file 12864_2015_1862_MOESM1_ESM.docx]

**Supplementary Table 1.** Summary of the studies on the effects of temperature on gene expression in fish

| Developmental stage | Methodology | Common name | Species | Treatment | Genes | Results | References | Temperature exposure | Sampling age |  |  |  |  |  |
| --- | --- | --- | --- | --- | --- | --- | --- | --- | --- | --- | --- | --- | --- | --- |
| During sex differentiation | Candidate gene approach | African catfish | *Clarias gariepinus* | Heat | *cyp19a1a* | F>M | Valenzuela et al., 2013 |  |  |  |  |  |  |  |
|  |  |  |  |  | *cyp19a1a* | HT<LT |  |  |  |  |  |  |  |  |
|  |  |  |  |  | *sf1* | F>M |  |  |  |  |  |  |  |  |
|  |  |  |  |  | *sox9* | F<M |  |  |  |  |  |  |  |  |
|  |  | Atlantic halibut | *Hippoglossus hippoglossus* | Heat | *wt1* | HT=LT | Valenzuela et al., 2013 |  |  |  |  |  |  |  |
|  |  | European sea bass | *Dicentrarchus labrax* | Heat | *cyp19a1a* | HT<LT | Blázquez et al., 1998 | 57-137 dpf | 21.4 months |  |  |  |  |  |
|  |  |  |  | Heat | *arb* | HT=LT | Blázquez et al., 2009 | 0-120 dpf | 120 dpf |  |  |  |  |  |
|  |  |  |  |  | *cyp11b* | HT<LT |  | 0-120 dpf | 120 dpf |  |  |  |  |  |
|  |  |  |  |  | *cyp19a1a* | HT<LT |  | 0-120 dpf | 195 dpf |  |  |  |  |  |
|  |  |  |  |  | *era* | HT=LT |  | 0-120 dpf | 120 dpf |  |  |  |  |  |
|  |  |  |  |  | *erb1* | HT=LT |  | 0-120 dpf | 120 dpf |  |  |  |  |  |
|  |  |  |  |  | *erb2* | HT=LT |  | 0-120 dpf | 120 dpf |  |  |  |  |  |
|  |  |  |  | Heat | *cyp19a1a* | HT<LT | Navarro-Martín et al., 2011 | 0-120 dph | 170 dph |  |  |  |  |  |
|  |  |  |  |  | *dax1* | HT=LT |  |  |  |  |  |  |  |  |
|  |  | Japanese flounder | *Paralichthys olivaceus* | Heat | *cyp19a1a* | HT<LT | Kitano et al., 1999 | 30-100 dph | 10-100 dph (biweekly) |  |  |  |  |  |
|  |  |  |  |  | *cyp19a1a* | HT<LT |  |  |  |  |  |  |  |  |
|  |  |  |  |  | *cyp19a1a* | HT<LT | Yamaguchi et al., 2007 | 30-100 dph | (biweekly) |  |  |  |  |  |
|  |  |  |  |  | *foxl2* | suppressed by heat |  | 30-100 dph | (biweekly) |  |  |  |  |  |
|  |  |  |  |  | *fshr* | suppressed by heat |  | 30-100 dph | (biweekly) |  |  |  |  |  |
|  |  | Nile tilapia | *Oreochromis niloticus* | Heat | *cyp19a1a* | HT<LT | D'Cotta et al., 2001 | 10-40 dpf | 18-26 dpf |  |  |  |  |  |
|  |  |  |  |  | *cyp19a1b* | HT<LT |  | 10-40 dpf | 18-26 dpf |  |  |  |  |  |
|  |  |  |  | Heat | *amh* | HT>LT | Poonlaphdecha et al., 2013 | 10 dph and onwards | 10 to 26 dpf |  |  |  |  |  |
|  |  |  |  |  | *dmrt1* | HT>LT |  | 10 dph and onwards | 10 to 26 dpf |  |  |  |  |  |
|  |  |  |  |  | *foxl2* | suppressed by heat |  | 10 dph and onwards | 10 to 26 dpf |  |  |  |  |  |
|  |  |  |  | Heat | *cyp19a1a* | F>M | Valenzuela et al., 2013 |  |  |  |  |  |  |  |
|  |  |  |  |  | *dax1* | F>M; after TSD F<M |  |  |  |  |  |  |  |  |
|  |  |  |  |  | *sf1* | F>M; after TSD F<M |  |  |  |  |  |  |  |  |
|  |  |  |  |  | *sox9* | F<M; mid-end TSD |  |  |  |  |  |  |  |  |
|  |  | Pejerrey | *Odontesthes bonaeriensis* | Heat | *amh* | HT>LT | Fernandino et al., 2008a | 0-56 dph | 0-42 dpf (biweekly) |  |  |  |  |  |
|  |  |  |  |  | *cyp19a1a* | HT<LT |  | 0-56 dph | 0-42 dpf (biweekly) |  |  |  |  |  |
|  |  |  |  | Heat | *cyp19a1a* | HT<LT | Valenzuela et al., 2013 | 0-56 dph | 0-42 dpf (biweekly) |  |  |  |  |  |
|  |  |  |  |  | *dmrt1* | HT>LT | Fernandino et al., 2008b | 0-56 dph | 0-42 dpf (biweekly) |  |  |  |  |  |
|  |  |  |  | Heat | *amh* | HT>LT | Hattori et al., 2009 | 0-126 dph | 28-35-49 dph |  |  |  |  |  |
|  |  |  |  |  | *cyp19a1a* | HT<LT |  | 0-126 dph | 28-35-49 dph |  |  |  |  |  |
|  |  |  |  | Heat | *cyp19a1a* | HT<LT | Karube et al., 2007 | 0-70 dph | 0-70 dph |  |  |  |  |  |
|  |  |  |  |  | *cyp19a1a* | HT<LT | Valenzuela et al., 2013 |  |  |  |  |  |  |  |
|  |  | Rainbow trout | *Oncorhynchus mykiss* | Heat | *wt1* | F<M; onset TSD | Valenzuela et al., 2013 |  |  |  |  |  |  |  |
|  |  |  |  |  | *cyp19a1a* | F>M |  |  |  |  |  |  |  |  |
|  |  |  |  |  | *dax1* | F>M; onset TSD |  |  |  |  |  |  |  |  |
|  |  |  |  |  | *sf1* | F<M: onset TSD; F>M: mid TSD |  |  |  |  |  |  |  |  |
|  |  |  |  |  | *sox9* | F<M |  |  |  |  |  |  |  |  |
|  |  | Zebrafish | *Danio rerio* | Heat | *cyp19a1a* | suppressed by heat | Uchida et al., 2004 | 15-25 dph | 40 dph |  |  |  |  |  |
| After sex differentiation is completed | Candidate gene approach | Atlantic halibut | *Hippoglossus hippoglossus* | Heat | *cyp19a1a* | HT<LT | Van Nes and Andersen., 2006 | 260 ddph | 260, 600, 1100 ddph |  |  |  |  |  |
|  |  | European sea bass | *Dicentrarchus labrax* | Heat | *cyp19a1a* | HT<LT | Navarro-Martín et al., 2011 | 0-120 dph | 330 dph |  |  |  |  |  |
|  |  |  |  |  | *cyp19a1b* | HT>LT |  | 260 ddph | 260, 600, 1100 ddph |  |  |  |  |  |
|  |  |  |  |  | *er1* | HT>LT |  | 260 ddph | 260, 600, 1100 ddph |  |  |  |  |  |
|  |  |  |  |  | *er2* | HT>LT |  | 260 ddph | 260, 600, 1100 ddph |  |  |  |  |  |
| Adult gonads | Candidate gene approach | Blue gourami | *Trichogaster trichopterus* | Heat | *gnrh3, igf1* | HT<LT; Females (male presence) affected under reproductive conditions | Levy et al., 2011 |  |  |  |  |  |  |  |
|  |  |  |  |  | *gnrh3, lh, gh* | HT<LT; Females affected under normal conditions |  | not specified | adults |  |  |  |  |  |
|  |  |  |  |  | *pacap, prp-pacap, gh, gonadotropins* | HT<LT; Females (no male presence) affected under reproductive conditions |  |  |  |  |  |  |  |  |
|  |  | Medaka | *Oryzias latipes* | Heat | *cyp19a1a* | suppressed by heat | Kitano et al., 2012 | 0-5 dph | adults |  |  |  |  |  |
|  |  |  |  |  | *dmrt1* | HT>LT | Hattori et al., 2007 | embryo development | 2-3 months |  |  |  |  |  |
|  |  |  |  |  |  |  |  |  |  |  |  |  |  |  |
|  | Transcriptomic analysis | Pejerrey | *Odontesthes bonaeriensis* | Heat | gsdf, *hsp90*, *cpa2*, *ctr*, *pretrypsinogen, etc…* | HT<LT | Fernandino et al., 2011 | 0-49 dph | 49 dph |  |  |  |  |  |
|  |  |  |  |  | *ndrg3, pen2, gadph, p2xr1, kininogen, etc…* | HT>LT |  | 0-49 dph | 49 dph |  |  |  |  |  |
|  |  |  |  |  | *wap56*, *aldoa* | HT>LT; warm acclimation |  | 0-49 dph | 49 dph |  |  |  |  |  |
|  |  |  |  |  |  |  |  |  |  |  |  |  |  |  |
| Abbreviations: ddph, degrees days post hatch; dpf, days post fertilization; dph, days post hatch; F, female; HT, high temperature; LT, low temperature; M, male | | | | | | | | | | |  |  |  |  |

**Supplementary Table 2.** Differentially expressed genes summary results

| HT vs. LT | # downregulated genes | # upregulated genes | # Total |
| --- | --- | --- | --- |
| Total annotated genes | 14,753 | 3,164 | 17,917 |
| Without repetitions | 4,789 | 1,360 | 6,149 |
| Significant genes (without repetitions) | 9 | 18 | 27 |

**Supplementary Table 3.**. Differentially expressed genes summary results

| Gene symbol | Gene name | Fold change (FC) | Adjusted *p*-value | Brief function description |
| --- | --- | --- | --- | --- |
| *adcy7* | Adenylate cyclase 7 | 1.688 | 3.01e-02 | Bound to the membrane and inhibitable by calcium. |
| *aptx* | Aprataxin | 1.801 | 4.53e-02 | DNA-binding protein involved in single- and soluble- strand DNA break repair and base excision repair. |
| *ca1* | Carbonic anhydrase | -3.173 | 4.53e-02 | Interconverts carbon dioxide and bicarbonate to maintain acid-base balance in blood and other tissues, and to help transport carbon dioxide out of tissues. |
| *ccbl1* | Cysteine conjugate beta-lyase cytoplasmic, isoform CRA-a | 1.324 | 4.53e-02 | Metabolism of cysteine conjugates of certain halogenated alkenes and alkanes. Can form reactive metabolites leading to nephrotoxicity, neurotoxicity and reproduction problems. |
| *cdc42ep3* | Cdc42 effector protein 3 | -1.986 | 2.41e-02 | Development of germ lines in flies and worms. May be a target of PUMILIO2 in human male testis. |
| *cg10623* | DmeI-CG10623 | 1.980 | 4.53e-02 | Homocysteine S-methyltransferase 1-like |
| *cldn3* | Claudin 3a | -4.265 | 2.08e-02 | Integral membrane protein and a component of tight junction strands. Major role in tight junction-specific obliteration of the intercellular space, through calcium-independent cell-adhesion activity. |
| *cmbl* | Carboxymethylene-butenolidase homolog | 2.377 | 4.53e-02 | Cysteine hydrolase. |
| *cno* | Cappuccino homolog | 1.369 | 4.53e-02 | Maternal effect loci: anteroposterior and dorsoventral patterns. May play a role in organelle biogenesis (lysosomes, melanosomes). |
| *cry-dash* | Cryptochrome DASH | 1.780 | 4.53e-02 | May have a photoreceptor function. Circadian clocks input, UV-damage repair (single-stranded DNA). Responsible of massive spawning during full moon in corals. |
| *ecm1* | Extracellular matrix protein 1 | -13.617 | 2.08e-02 | Involved in endochondral bone formation as negative regulator of bone mineralization. Also, stimulates the proliferation of endothelial cells and promotes angiogenesis. Present in maturing trout ovaries and in a higher concentration when compared to testis or pre-spawning ovaries. |
| *gnai1* | Guanine nucleotide-binding protein G(i) subunit alpha-1 | 1.913 | 2.58e-02 | Modulators or transducers in various transmembrane signaling systems. Involved in hormonal regulation of adenylate cyclase by inhibiting the cyclase in response to beta-adrenergic stimuli. May also play a role in cell division. |
| *gng13* | Guanine nucleotide binding protein G(I)/G(S)/G(O) subunit gamma-13 | -1.795 | 2.08e-02 | Modulators or transducers in various transmembrane signaling systems. Regulation of melatonin receptors in the brain. |
| *gtf3c1* | General transcription factor 3C polypeptide 1 | 2.697 | 2.08e-02 | Required for RNA polymerase III-mediated transcription. |
| *hdac11* | Class 4 histone deacetylase 11 protein | 1.647 | 2.08e-02 | Deacetylates lysine residues on the N-terminal part of the core histones (H2A, H2B, H3 and H4). Giving a tag for epigenetic repression, thus playing an important role in transcriptional regulation, cell cycle progression and developmental events. |
| *igf1* | Insulin-like growth factor I | -7.119 | 4.53e-02 | Related to insulin with a high growth-promoting activity. Sex steroids, growth hormone and igf1 play a role in neuroendocrine and metabolic regulation of puberty. |
| *kin* | DNA/RNA binding protein KIN17 | 1.422 | 2.86e-02 | Involved in DNA replication and cellular response to DNA damage. May form a bridge between DNA replication and repair mediated by high molecular weight complexes. May also play a role in illegitimate recombination and regulation of gene expression. |
| *nefm* | Neurofilament medium polypeptide | 1.472 | 2.08e-02 | Involved in the maintenance of neuronal caliber. They may also play a role in intracellular transport to axons and dendrites. |
| *pddc1* | Parkinson disease 7 domain containing 1 | 1.666 | 2.08e-02 | Regulation of cell cycle arrest and apoptosis following double strand DNA breaks. |
| *prpf40a* | Pre-mRNA processing factor 40 homolog A | 2.00 | 2.08e-02 | Plays a role in the regulation of cell morphology and cytoskeletal organization, in cell shape and migration control; and may be involved in pre-mRNA splicing. |
| *smtn1* | Smoothelin 1 | -4.881 | 2.08e-02 | A key factor governing sexual development and pregnancy. Induces adaptations in smooth and striated muscle. Produces infertility in KO mice phenotype. |
| *ston1-gtf2a1l* | Protein STON1-GTF2A | 1.667 | 4.53e-02 | Not determined yet. |
| *tep1* | Telomerase protein component 1 | 1.894 | 2.08e-02 | Telomerase activity which catalyzes the addition of new telomeres on the chromosome ends. Its activity is related to a DNA-methylation increase and to a telomeric transcription reduction. |
| *tmpo* | Lamina-associated polypeptide 2, isoform alpha | 2.182 | 2.58e-02 | May maintain the structural organization of the nuclear envelope. May be involved in the structural organization of the nucleus and in the post-mitotic nuclear assembly. *tmpo1* and *tmpo5* may play a role in T-cell development and function. |
| *tnnI* | Troponin-I isoform 2 | 1.695 | 2.05e-02 | Thin filament regulatory complex which confers calcium-sensitivity to striated muscle actomyosin ATPase activity. Controls ovulatory contraction of non-striated actomyosin network in gonads. |
| *wisp1* | WNT1 inducible signaling pathway protein 1 | -1.684 | 3.44e-02 | Associated with cell survival, p53-mediated apoptosis attenuation in response to DNA damage and with the up-regulation of the anti-apoptotic Bcl-X(L) protein. Sex hormone signaling in endometrial homeostasis. |
| *yes1* | Tyrosine protein kinase Yes | -1.356 | 4.53e-02 | Involved in the regulation of cell growth and survival, apoptosis, cell-cell adhesion, cytoskeleton remodeling, differentiation and cell cycle progression (regulating the G1 phase by cdk4 phosphorylation and the G2/M progression and cytokinesis). |

Note: Function description taken from Genecards ([http://www.genecards.org](http://www.genecards.org/)) and Uniprot ([http://www.uniprot.org](http://www.uniprot.org/)) databases.

**Supplementary Table 4.** Fisher’s exact test with multiple testing correction of FDR depicting the over-represented GO terms

| GO Term | Name | Type | FDR | p-value | # in test group | # in reference group | # non annotated test group | # non annotated reference group | Over/Under |
| --- | --- | --- | --- | --- | --- | --- | --- | --- | --- |
| GO:0003008 | System process | Biological process | 1.8e-02 | 5.1e-06 | 9 | 342 | 19 | 6,672 | Over |
| GO:0051970 | Negative regulation of transmission of nerve impulse | Biological process | 1.8e-02 | 6.6e-06 | 3 | 7 | 25 | 7,007 | Over |
| GO:0050805 | Negative regulation of synaptic transmission | Biological process | 1.8e-02 | 6.6e-06 | 3 | 7 | 25 | 7,007 | Over |
| GO:0031645 | Negative regulation of neurological system process | Biological process | 1.8e-02 | 6.6e-06 | 3 | 7 | 25 | 7,007 | Over |
| GO:0007193 | Adenylate cyclase-inhibiting G-protein coupled receptor signaling pathway | Biological process | 2.6e-02 | 1.2e-05 | 3 | 9 | 25 | 7,005 | Over |

**Supplementary Table 5.** Summary of the KEGG pathways from the up- and downregulated gene list

| Pathway name | # sequences | # enzymes | Genes involved | Up/Down |
| --- | --- | --- | --- | --- |
| Alanine, aspartate and glutamate metabolism | 1 | 1 | *ccbl1* | Up |
| Cysteine and methionine metabolism | 2 | 2 | *cg10623 + ccbl1* | Up |
| Glyoxylate and dicarboxylate metabolism | 1 | 1 | *aptx* | Up |
| Inositol phosphate metabolism | 1 | 2 | *teP1* | Up |
| Nitrogen metabolism | 1 | 1 | *ca1* | Down |
| Phenylalanine metabolism | 1 | 1 | *ccbl1* | Up |
| Phenylalanine, tyrosine and tryptophan biosynthesis | 1 | 1 | *ccbl1* | Up |
| Phosphatidylinositol signaling system | 1 | 2 | *tep1* | Up |
| Purine metabolism | 1 | 1 | *adcy7* | Up |
| T cell receptor signaling pathway | 1 | 1 | *yes1* | Down |
| Tropane, piperidine and pyridine alkaloid biosynthesis | 1 | 1 | *ccbl1* | Up |
| Tryptophan metabolism | 1 | 1 | *ccbl1* | Up |
| Tyrosine metabolism | 1 | 1 | *ccbl1* | Up |

**Supplementary Table 6.** Microarray versus qRT-PCR fold change results for 15 selected reproduction-related genes

|  | Microarray | | qRT-PCR | |
| --- | --- | --- | --- | --- |
| Genes | Fold change (FC) | Adjusted *P*-value | Fold change (FC) | *P*-value |
| *amh* | 6.36 | 0.288 | 1.40 | 0.236 |
| *aqp1* | -5.47 | 0.169 | 0.11 | 0.023* |
| *col18a1* | 2.56 | 0.259 | 0.59 | 0.566 |
| *cyp19a1a* | -1.70 | 0.740 | 0.22 | 0.039* |
| *cyp19a1b* | 1.06 | 0.756 | 0.97 | 0.388 |
| *dmrt1* | 1.73 | 0.339 | 5.19 | 0.017* |
| *gnrh* | -1.08 | 0.965 | 13.61 | 0.083 |
| *igf1* | -7.12 | 0.045* | 0.90 | 0.901 |
| *mettl22* | 1.17 | 0.567 | 2.26 | 0.134 |
| *prl* | -1.02 | 0.974 | 3.24 | 0.039 |
| *sox17* | -1.20 | 0.724 | 0.87 | 0.528 |
| *star* | 1.56 | 0.401 | 2.75 | 0.036* |
| *tesc* | 1.09 | 0.900 | 1.06 | 0.219 |
| *vasa* | - | - | 12.74 | 0.057 |
| *wisp1* | -1.39 | 0,020* | -2.08 | 0.064 |

**Supplementary Table 7.** Epigenetic regulatory mechanisms- related genes implicated in sex differentiation as discussed in Piferrer (2013) that are present in the European sea bass custom-made microarray used in this study

|  |  |  | Upregulated | | Downregulated | |
| --- | --- | --- | --- | --- | --- | --- |
| Gene type | Gene name | Gene abbreviation | FC | Adjusted p-value | FC | Adjusted p-value |
| Heat shock proteins | heat responsive protein 12 | *hrsP12* | 1,50 | 0,536 | - | - |
|  | heat shock factor binding protein 1 | *hsbP1* | - | - | -1,13 | 0,706 |
|  | heat shock protein 10 | *hsPa10* | - | - | -1,15 | 0,869 |
|  | heat shock protein 14 | *hsPa14* | 1,20 | 0,694 | - | - |
|  | heat shock protein 4 | *hsPa4* | - | - | -1,23 | 0,689 |
|  | heat shock protein 60 | *hsPa60* | - | - | -1,15 | 0,824 |
|  | heat shock protein 70 | *hsPa70* | 1,29 | 0,513 | - | - |
|  | heat shock protein 70 binding protein | *hsPa70bP* | - | - | 1,05 | 0,973 |
|  | heat shock protein 70 isoform 3 | *hsPa70* | 1,20 | 0,893 | - | - |
|  | heat shock protein 71 | *hsPa71* | 1,16 | 0,897 | - | - |
|  | heat shock protein 90B | *hsPa90b* | 1,63 | 0,351 | - | - |
|  | heat shock protein transcription factor 2 binding protein | *hsf2bP* | - | - | 1,0 | 0,906 |
| Histone deacetylases | histone deacetylase | *hdac* | - | - | -1,1 | 0,949 |
|  | histone deacetylase 11 | *hdac11* | 1,64 | 0,424 | - | - |
|  | histone deacetylase 7 isoform D | *hdac7* | - | - | 1,1 | 0,520 |
| Dicer | dicer 1 (DCR-1 homolog) | *dicer1* | - | - | -1,1 | 0,384 |
| Jarid (Jumonji) | Histone demethylase JARID1B | *kdm5b* | 1,2 | 0,766 | - | - |
|  | Protein Jumonji | *jarid2a* | - | - | -1,1 | 0,762 |
| JmjC-containing H3K9 demethylase | Lysine-specific demethylase 3a | *kdm3a* | 1,2 | 0,647 | - | - |
|  | Lysine-specific demethylase 3b | *kdm3b* | 1,3 | 0,259 | - | - |
|  | Lysine-specific demethylase 6b | *kdm6b* | - | - | 1,1 | 0,973 |
| Polycomb | Polycomb group RING finger protein 2 | *pcgf2* | 1,4 | 0,193 | - | - |
|  | Polycomb group RING finger protein 5 | *pcgf5* | 1,3 | 0,420 | - | - |
|  | Polycomb group RING finger protein 6 | *pcgf6* | 1,3 | 0,446 | - | - |
| Thritorax suppressor | suppressor of zeste 12 homolog | *suz12* | - | - | 1,0 | 0,934 |
| Histone-lysine N-methyltransferase | Euchromatic histone-lysine N-methyltransferase 2 | *ehmt2* | 1,7 | 0,127 | - | - |

**Supplementary Table 8.** Comparison between the microarray results of this study and those reported in the literature on effects of temperature at the transcriptomic level (Gracey et al., 2004; Podrabsky and Somero, 2004; Cossins et al., 2006; Vergauwen et al., 2010; Chojnowski and Braun, 2012)

| Probe ID | Description | Median | adjusted p-value |
| --- | --- | --- | --- |
| CUST_21190_PI416070213 | 40S ribosomal protein Sa-like protein | 1,0638 | 9,62E-01 |
| CUST_4351_PI416070213 | acetyl-CoA acetyltransferase 2 | 1,0840 | 9,66E-01 |
| CUST_12556_PI416070213 | acidic leucine-rich nuclear phosphoprotein 32 family member A (PHAPI) | -1,0522 | 9,66E-01 |
| CUST_3520_PI416070213 | ADP-ribosylation factor-like 1 | -1,1735 | 5,88E-01 |
| CUST_1012_PI416070213 | amh gene for anti-Müllerian hormone | 4,8979 | 9,90E-01 |
| CUST_20077_PI416070213 | anti-Müllerian hormone | -1,0522 | 2,68E-01 |
| CUST_5686_PI416070213 | antizyme inhibitor 1 (AZI) | -1,2325 | 9,72E-01 |
| CUST_11722_PI416070213 | apolipoprotein A-I binding protein | 1,0923 | 2,10E-01 |
| CUST_4720_PI416070213 | apolipoprotein A-I binding protein, isoform CRA_a | -3,8293 | 8,03E-01 |
| CUST_8305_PI416070213 | apolipoprotein AI precursor | -1,0262 | 4,67E-01 |
| CUST_14611_PI416070213 | apolipoprotein A-IV3 | 1,0615 | 9,87E-01 |
| CUST_2026_PI416070213 | apolipoprotein B | -1,6506 | 9,42E-01 |
| CUST_2278_PI416070213 | apolipoprotein E | -5,4703 | 7,69E-01 |
| CUST_12361_PI416070213 | aquaporin 1 | 1,3650 | 1,69E-01 |
| CUST_14552_PI416070213 | aquaporin 8 | 1,0026 | 7,86E-02 |
| CUST_15718_PI416070213 | arginase II | -1,0562 | 9,96E-01 |
| CUST_13558_PI416070213 | arginine-rich protein specific kinase 1 | 1,0688 | 9,44E-01 |
| CUST_12544_PI416070213 | ATP synthase | -1,0863 | 6,72E-01 |
| CUST_3988_PI416070213 | ATP synthase a chain | 1,1256 | 9,07E-01 |
| CUST_10513_PI416070213 | ATP/GTP binding protein-like 4 | 1,7382 | 5,30E-01 |
| CUST_2071_PI416070213 | ATPase, Na+/K+ transporting, beta 1 | -1,1077 | 7,12E-01 |
| CUST_5095_PI416070213 | basic transcription factor 3 isoform B | 1,2555 | 8,58E-01 |
| CUST_9550_PI416070213 | basic transcription factor 3-like 4 | -1,0223 | 6,38E-01 |
| CUST_6508_PI416070213 | beta-catenin | -1,9358 | 9,82E-01 |
| CUST_17692_PI416070213 | betaine homocysteine S-methyltansferase | -1,2578 | 2,45E-01 |
| CUST_14122_PI416070213 | btf3l4 protein | 1,3894 | 8,05E-01 |
| CUST_12253_PI416070213 | C1q-like adipose specific protein | -1,1889 | 7,65E-01 |
| CUST_18358_PI416070213 | calmodulin (CaM) | 1,0179 | 8,61E-01 |
| CUST_1480_PI416070213 | calreticulin | -1,1267 | 9,92E-01 |
| CUST_110_PI416268254 | carbonic anhydrase | -2,6397 | 7,93E-01 |
| CUST_670_PI416070213 | carboxypeptidase A1 | -1,0225 | 8,97E-01 |
| CUST_21325_PI416070213 | cardiac myosin light chain-1 | -1,1255 | 9,76E-01 |
| CUST_5875_PI416070213 | cathepsin H precursor | -1,1023 | 9,93E-01 |
| CUST_16342_PI416070213 | cds2 protein | -1,3620 | 7,54E-01 |
| CUST_1666_PI416070213 | chain A, Refined Solution Structure Of Human Profilin I | -1,3922 | 9,70E-01 |
| CUST_7264_PI416070213 | cofilin 2, muscle, isoform CRA_b | 1,3753 | 5,99E-01 |
| CUST_1303_PI416070213 | cofilin protein | -1,1150 | 4,98E-01 |
| CUST_139_PI416070213 | cold-shock domain protein mRNA | 1,0075 | 9,40E-01 |
| CUST_14164_PI416070213 | copper chaperone for superoxide dismutase | 1,0576 | 6,00E-02 |
| CUST_8065_PI416070213 | copper/zinc superoxide dismutase | -1,0237 | 7,03E-01 |
| CUST_2563_PI416070213 | creatine kinase mitochondrial isoform | -1,1363 | 9,95E-01 |
| CUST_4120_PI416070213 | cyclin G1 | -1,3054 | 9,85E-01 |
| CUST_30_PI416268254 | cytochrome b | -1,3147 | 4,25E-01 |
| CUST_7084_PI416070213 | cytochrome c oxidase subunit Vb precursor | 1,0107 | 8,53E-01 |
| CUST_6961_PI416070213 | cytochrome c oxidase subunit VIb isoform 1 | -1,0921 | 8,53E-01 |
| CUST_1_PI416268254 | cytochrome P450 | -1,2184 | 9,70E-01 |
| CUST_535_PI416070213 | cytochrome P450 aromatase B (cyp19b) | -1,1207 | 9,42E-01 |
| CUST_16732_PI416070213 | cytoplasmic polyadenylation element binding protein | -1,1492 | 7,70E-01 |
| CUST_6385_PI416070213 | cytosolic malate dehydrogenase thermolabile form | 1,0476 | 3,05E-01 |
| CUST_6439_PI416070213 | dazap1 protein | -1,2408 | 4,49E-01 |
| CUST_3067_PI416070213 | dazap2-like protein | 1,0065 | 9,47E-01 |
| CUST_12025_PI416070213 | death associated protein 1a | 1,0243 | 7,02E-01 |
| CUST_17401_PI416070213 | delta-9-desaturase 1 | -1,0487 | 3,08E-01 |
| CUST_21223_PI416070213 | dihydrolipoamide dehydrogenase | -1,1800 | 9,52E-01 |
| CUST_17317_PI416070213 | dihydropyrimidine dehydrogenase | 1,0199 | 8,61E-01 |
| CUST_12487_PI416070213 | DM-related transcriptional factor Dmrt2b | 1,2953 | 9,55E-01 |
| CUST_10631_PI416070213 | dmrt 1 | 1,2080 | 4,91E-01 |
| CUST_3052_PI416070213 | early growth response 1 | -1,0577 | 9,40E-01 |
| CUST_10501_PI416070213 | eif1ad protein | -1,3994 | 9,67E-01 |
| CUST_10678_PI416070213 | elastase 1 precursor | -1,4463 | 9,22E-01 |
| CUST_8971_PI416070213 | elastase-like serine protease | -1,5505 | 8,89E-01 |
| CUST_15238_PI416070213 | elastin microfibril interfacer 2 | -2,1761 | 2,66E-01 |
| CUST_21118_PI416070213 | elongation factor 1 alpha isoform 2 | 1,1757 | 2,53E-01 |
| CUST_16189_PI416070213 | ependymin | -1,0285 | 9,04E-01 |
| CUST_12778_PI416070213 | ependymin-2 precursor | -1,0623 | 9,27E-01 |
| CUST_9475_PI416070213 | eukaryotic translation initiation factor 2B | -1,0340 | 9,73E-01 |
| CUST_18418_PI416070213 | eukaryotic translation initiation factor 3 | -1,0658 | 9,65E-01 |
| CUST_13579_PI416070213 | F-box only protein 2 | -1,1159 | 9,77E-01 |
| CUST_8251_PI416070213 | ferritin-H subunit | -1,5972 | 8,49E-01 |
| CUST_21229_PI416070213 | filamin A interacting protein 1 | -1,3786 | 5,06E-01 |
| CUST_2752_PI416070213 | filamin A, alpha | -3,4245 | 4,92E-01 |
| CUST_11212_PI416070213 | galectin | -1,8040 | 5,49E-01 |
| CUST_16003_PI416070213 | galectin 8 | 1,0283 | 9,18E-01 |
| CUST_7216_PI416070213 | galectin like protein | -1,1072 | 8,48E-01 |
| CUST_6142_PI416070213 | gelatinase | 1,0543 | 9,73E-01 |
| CUST_2614_PI416070213 | glutathione S-transferase, theta 3 | -2,3106 | 4,18E-01 |
| CUST_16666_PI416070213 | glycine dehydrogenase (decarboxylating) | 1,4019 | 5,55E-01 |
| CUST_13426_PI416070213 | granulin-a | 1,4162 | 6,66E-01 |
| CUST_12714_PI416070213 | granulin-like peptide | -1,0972 | 9,22E-01 |
| CUST_3412_PI416070213 | GTP-binding nuclear protein Ran (GTPase Ran) | 1,0162 | 9,92E-01 |
| CUST_2419_PI416070213 | heat shock cognate 70 | -1,0475 | 9,31E-01 |
| CUST_17674_PI416070213 | heat shock cognate 71 | 1,1065 | 9,37E-01 |
| CUST_7477_PI416070213 | heat shock factor binding protein 1 | -1,0850 | 8,72E-01 |
| CUST_6343_PI416070213 | heat shock protein 10 | -1,1418 | 8,95E-01 |
| CUST_5653_PI416070213 | heat shock protein 70 binding protein | 1,0576 | 9,66E-01 |
| CUST_18040_PI416070213 | heat shock protein 90 beta | 1,0685 | 9,54E-01 |
| CUST_15709_PI416070213 | heterogeneous nuclear ribonucleoprotein G | 1,2192 | 6,96E-01 |
| CUST_7765_PI416070213 | high density lipoprotein (HDL) binding protein | -1,1989 | 9,08E-01 |
| CUST_11980_PI416070213 | high mobility group protein | -1,0976 | 8,69E-01 |
| CUST_18037_PI416070213 | High-mobility group box 1 | -1,2261 | 4,73E-01 |
| CUST_10831_PI416070213 | high-mobility group box 2 | -1,1422 | 8,93E-01 |
| CUST_17146_PI416070213 | high-mobility group protein 2-like 1 | -1,0172 | 9,87E-01 |
| CUST_13807_PI416070213 | Histone H2B | -1,0702 | 9,85E-01 |
| CUST_8773_PI416070213 | histone H3 | -1,0883 | 8,62E-01 |
| CUST_283_PI416070213 | HMG-CoA reductase mRNA | 1,2728 | 9,83E-01 |
| CUST_3016_PI416070213 | hsp47 | -1,1294 | 8,36E-01 |
| CUST_10309_PI416070213 | isocitrate dehydrogenase 1 (NADP+) | -1,0688 | 3,18E-01 |
| CUST_17161_PI416070213 | isocitrate dehydrogenase 3 (NAD+) alpha | -1,0518 | 9,41E-01 |
| CUST_5272_PI416070213 | isocitrate dehydrogenase 3 (NAD+) gamma isoform | 1,0149 | 9,57E-01 |
| CUST_13297_PI416070213 | isocitrate dehydrogenase 3, beta subunit isoform | 1,3798 | 9,66E-01 |
| CUST_8839_PI416070213 | junB protein | -1,0054 | 9,64E-01 |
| CUST_15787_PI416070213 | kinesin-like protein KIF1B | -1,1594 | 9,91E-01 |
| CUST_8515_PI416070213 | lecithin cholesterol acyltransferase | 1,0038 | 7,53E-01 |
| CUST_10357_PI416070213 | leucine rich repeat containing 42 | -1,1614 | 9,95E-01 |
| CUST_4639_PI416070213 | malate dehydrogenase | -1,0179 | 6,37E-01 |
| CUST_16465_PI416070213 | malic enzyme 3, NADP(+)-dependent | 1,0512 | 9,76E-01 |
| CUST_9052_PI416070213 | manganese superoxide dismutase | 1,1171 | 9,74E-01 |
| CUST_11593_PI416070213 | mdh1b protein | 1,1124 | 3,60E-01 |
| CUST_19768_PI416070213 | microsomal glutathione S-transferase | -1,0105 | 9,46E-01 |
| CUST_11806_PI416070213 | microsomal glutathione S-transferase 2 | 1,1775 | 9,86E-01 |
| CUST_9199_PI416070213 | mitochondrial ATP synthase gamma-subunit | -1,0344 | 8,91E-01 |
| CUST_18255_PI416070213 | mitochondrial creatine kinase | 1,0804 | 9,04E-01 |
| CUST_4891_PI416070213 | mitochondrial ornithine aminotransferase | -1,0246 | 9,47E-01 |
| CUST_3516_PI416070213 | mitochondrial uncoupling protein 3 | 1,0542 | 9,76E-01 |
| CUST_6304_PI416070213 | mitosis-specific chromosome segregation protein SMC1 homolog | -1,2270 | 9,27E-01 |
| CUST_18190_PI416070213 | muc2 protein | -1,0744 | 7,77E-01 |
| CUST_12637_PI416070213 | mucin 2, oligomeric mucus/gel-forming, isoform CRA_a | -1,1538 | 7,88E-01 |
| CUST_21289_PI416070213 | myosin heavy chain | -1,3612 | 9,65E-01 |
| CUST_1252_PI416070213 | myosin light chain 1 | -1,0918 | 9,86E-01 |
| CUST_7768_PI416070213 | NADH dehydrogenase subunit 4 | -3,1728 | 7,13E-01 |
| CUST_21013_PI416070213 | obscurin | 1,1429 | 7,65E-01 |
| CUST_1846_PI416070213 | ornithine aminotransferase | -1,0326 | 9,94E-01 |
| CUST_11446_PI416070213 | ornithine decarboxylase antizyme | 1,0080 | 9,78E-01 |
| CUST_1201_PI416070213 | orphan nuclear receptor DAX2 (NR0B1b) gene | -1,0978 | 9,76E-01 |
| CUST_17446_PI416070213 | oxidoreductase NAD-binding domain containing 1 | -1,0357 | 9,01E-01 |
| CUST_1459_PI416070213 | pc4 and sfrs1-interacting protein | -1,2041 | 8,50E-01 |
| CUST_9442_PI416070213 | pdia4 protein | 1,1513 | 9,42E-01 |
| CUST_2647_PI416070213 | phosphoenolpyruvate carboxykinase | 1,1043 | 7,40E-01 |
| CUST_18646_PI416070213 | postmeiotic segregation increased 2 (pms2) | -2,7459 | 6,78E-01 |
| CUST_1597_PI416070213 | probable Bax inhibitor 1 (BI-1) | -1,1457 | 9,66E-01 |
| CUST_10171_PI416070213 | profilin 2 like | 1,0323 | 9,42E-01 |
| CUST_2938_PI416070213 | proteasome (prosome, macropain) 26S subunit, non-ATPase, 10 | -1,0459 | 9,80E-01 |
| CUST_7993_PI416070213 | proteasome (prosome, macropain) subunit, alpha | 1,0707 | 9,46E-01 |
| CUST_13930_PI416070213 | proteasome (prosome, macropain) subunit, beta type, 5 | 1,1885 | 9,33E-01 |
| CUST_2773_PI416070213 | ptp-IV1b, PTP-IV1 gene product | -1,0839 | 9,96E-01 |
| CUST_17542_PI416070213 | ptprd protein | -1,0032 | 6,66E-01 |
| CUST_15433_PI416070213 | ptprf interacting protein, binding protein 1 | -1,1672 | 7,56E-01 |
| CUST_14260_PI416070213 | putative pyruvate dehydrogenase phosphatase isoenzyme 2 | -1,0551 | 8,52E-01 |
| CUST_16117_PI416070213 | pyruvate dehydrogenase (lipoamide) beta | 1,0572 | 6,31E-01 |
| CUST_1510_PI416070213 | pyruvate dehydrogenase E1 component subunit | -1,1418 | 8,81E-01 |
| CUST_10711_PI416070213 | pyruvate kinase | -1,0835 | 9,38E-01 |
| CUST_19390_PI416070213 | quiescin Q6 sulfhydryl oxidase 1 isoform a | 1,0826 | 9,49E-01 |
| CUST_18058_PI416070213 | ran binding protein 10 | 1,0305 | 9,93E-01 |
| CUST_7159_PI416070213 | ran binding protein 3 isoform RANBP3-b | -1,0069 | 9,52E-01 |
| CUST_2284_PI416070213 | ran-binding protein 7 | -1,0678 | 9,94E-01 |
| CUST_7891_PI416070213 | ranbp1 protein | 1,0132 | 9,02E-01 |
| CUST_4411_PI416070213 | RNA-binding protein 1 | -1,1543 | 6,03E-01 |
| CUST_16006_PI416070213 | rrm1 protein | -1,1065 | 8,24E-01 |
| CUST_21007_PI416070213 | sarcoglycan, gamma | 1,2231 | 8,88E-01 |
| CUST_19216_PI416070213 | serine carboxypeptidase 1 | -1,1060 | 9,78E-01 |
| CUST_12704_PI416070213 | skeletal muscle myosin heavy chain | -1,0691 | 1,09E-01 |
| CUST_20632_PI416070213 | slow myosin heavy chain 3 | -1,0207 | 8,61E-02 |
| CUST_14320_PI416070213 | small nuclear ribonucleoprotein D2-like protein | 1,2830 | 9,89E-01 |
| CUST_1174_PI416070213 | small nuclear ribonucleoprotein E-like mRNA | 3,2432 | 4,22E-01 |
| CUST_14152_PI416070213 | sodium potassium ATPase alpha subunit | 1,0153 | 5,77E-01 |
| CUST_10027_PI416070213 | solute carrier family | -1,6132 | 9,56E-01 |
| CUST_16327_PI416070213 | solute carrier family 3, member 2 | -1,0341 | 4,23E-01 |
| CUST_19864_PI416070213 | splicing factor 3a, subunit 1, 120kDa isoform 1 | 1,3642 | 5,81E-01 |
| CUST_15601_PI416070213 | splicing factor 3b, subunit 1 isoform 1 | 1,2465 | 8,90E-01 |
| CUST_3085_PI416070213 | splicing factor arginine/serine-rich 3 | -1,1149 | 9,27E-01 |
| CUST_12128_PI416070213 | T-complex protein 1 subunit alpha | 1,1564 | 4,64E-01 |
| CUST_20779_PI416070213 | tcp1 protein | -1,1055 | 9,35E-01 |
| CUST_5194_PI416070213 | tcp1-beta | -1,0641 | 9,33E-01 |
| CUST_10183_PI416070213 | tomm20 | 1,0960 | 8,60E-01 |
| CUST_12044_PI416070213 | transaldolase | 1,0797 | 8,53E-01 |
| CUST_17176_PI416070213 | transcription factor SOX-8 | 1,3126 | 9,44E-01 |
| CUST_12057_PI416070213 | translation initiation factor eIF-2B precursor | -1,0425 | 9,63E-01 |
| CUST_21553_PI416070213 | tubulin alpha 6 | 1,0143 | 8,21E-01 |
| CUST_7112_PI416070213 | tubulin, alpha, ubiquitous | -1,1704 | 6,22E-01 |
| CUST_17711_PI416070213 | tubulin, delta 1, isoform CRA_c | 1,2051 | 9,28E-01 |
| CUST_13015_PI416070213 | tubulin, gamma complex associated protein 2 | 1,1493 | 4,98E-01 |
| CUST_88_PI416070213 | U2AF1-RS2 | 1,2263 | 7,32E-01 |
| CUST_6817_PI416070213 | ubiquitin conjugating enzyme E2A | 1,1512 | 7,07E-01 |
| CUST_4522_PI416070213 | UDP-N-acetylglucosamine transferase subunit | -1,0196 | 9,85E-01 |
| CUST_3094_PI416070213 | vacuolar ATP synthase 16 kDa proteolipid subunit-like protein | -1,0186 | 9,81E-01 |
| CUST_13924_PI416070213 | wnt1 inducible signaling pathway protein 1 | -1,6458 | 6,48E-02 |

**Supplementary Table 9.** Gene abbreviation glossary

| Gene abbreviations | Gene name |
| --- | --- |
| *adcy7* | Adenylate cyclase 7 |
| *amh* | Anti-Müllerian hormone |
| *aptx* | Aprataxin |
| *aqp1* | Aquaporin 1 |
| *ca1* | Carbonic anhydrase 1 |
| *ccbl1* | Cysteine conjugate beta-lyase cytoplasmic, isoform CRA_a |
| *cdc42ep3* | Cdc42 efector protein 3 |
| *cg10623* | DmeI-CG10623 |
| *cldn3* | Claudin 3 |
| *cmbl* | Carboxymethylenebutenolidase homolog |
| *cno* | Cappuccino homolog |
| *col18a1* | Collagen alpha-1 (XVIII) chain |
| *cry-dash* | Cryptochrome DASH |
| *cyp19a1a* | Cytochrome P450, family 19, subfamily A, polypeptide 1a |
| *cyp19a1b* | Cytochrome P450, family 19, subfamily A, polypeptide 1b |
| *dicer1* | Endoribonuclease Dicer |
| *dmrt1* | Doublesex- and mab-3- related transcription factor I |
| *ecm1* | Extracellular matrix protein 1 |
| *ehmt2* | Euchromatic histone-lysine N-methyltransferase 2 |
| *gnai1* | Guanine nucleotide-binding protein G(i) subunit alpha-1 |
| *gng13* | Guanine nucleotide-binding protein G(I)/G(S)/G(O) subunit gamma-13 |
| *gnrh* | Gonadotropin-releasing hormone |
| *nr3c1* | Glucocorticoid receptor |
| *gtf3c1* | General transcription factor 3C polypeptide 1 |
| *hdac11* | Histone deacetylase 11 |
| *hsd11b1* | 11β-hydroxysteroid dehydrogenase |
| *igf1* | Insulin-like growth factor I |
| *jarid2a* | Protein Jumonji |
| *kin* | DNA/RNA-binding protein KIN17 |
| *mettl22* | Methyltransferase-like protein 22 |
| *nefm* | Neurofilament medium polypeptide |
| *pcgf2* | Polycomb group ring finger 2 |
| *pddc1* | Parkinson disease 7 domain-containing protein 1 |
| *prl* | Prolactin |
| *prpf40a* | Pre-mRNA-processing factor 40 homolog A |
| *r18S* | r18S |
| *smtn1* | Smoothelin 1 |
| *sox17* | HMG-box transcription factor SOX17 |
| *star* | Steroidogenic acute regulatory protein |
| *ston1-gtf2a1l* | Protein STON1-GTF2A |
| *suz12* | Suppressor of zeste 12 homolog |
| *tep1* | Telomerase protein component 1 |
| *tesc* | Tescalcin |
| *tmpo* | Lamina-associated polypeptide 2, isoform alpha |
| *tnnI* | Troponin I |
| *vasa* | Vasa protein |
| *wisp1* | WNT1 inducible signaling pathway protein 1 |
| *yes1* | Tyrosine protein kinase Yes |

**Supplementary Table 10.** Quantitative QRT-PCR primer characteristics

| Gene | Gene abbreviation | Primer name | Primer sequence (5’→3’) | Efficiency (*E*) | Slope | R^2^ |
| --- | --- | --- | --- | --- | --- | --- |
| 11β-hydroxysteroid dehydrogenase | *hsd11b1* | hsd11b1-Fw | CCTGGCAGCATATGGAGCAT | 2.26 | -2.82 | 0.92 |
|  |  | hsd11b1-Rv | TACTGGTGCGACCTGTCCTA |  |  |  |
| Anti-Müllerian hormone | *amh* | amh-Fw | TGCAGAGCAAAGCCTGAAAG | 2.10 | -3.09 | 0.99 |
|  |  | amh-Rv | TCAACGGGGAACAAAGACAA |  |  |  |
| Aquaporin 1 | *aqp1* | aqua-Fw | GCCAGATCAGCGTGTTCAAG | 2.27 | -2.89 | 0.98 |
|  |  | aqua-Rv | ACAGCACCAGCTGGAAGGTT |  |  |  |
| Carbonic anhydrase 1 | *ca1* | ca1-Fw | TGCCATAGTTGCTAACGCAC | 2.22 | -2.88 | 0.97 |
|  |  | ca1-Rv | CTCATGGGACAGCCCTAACA |  |  |  |
| Cdc42 efector protein 3 | *cdc42ep3* | cdc42ep3-Fw | AGAGATCCTGCAGATGGACG | 2.15 | -3.00 | 0.99 |
|  |  | cdc42ep3-Rv | TGTGTTGCTGTTCAGGCTTC |  |  |  |
| Collagen alpha-1 (XVIII) chain | *col18a1* | col-Fw | AACTGCGACTCGGATCCTCA | 2.01 | -3.17 | 0.98 |
|  |  | col-Rv | TATCCGGGTCTGCTCCACTG |  |  |  |
| Cryptochrome DASH | *cry-dash* | cry-dash-Fw | GTTTGGGACAAAGCGTGCTA | 2.29 | -2.65 | 0.96 |
|  |  | cry-dash-Rv | CTGTCTGTTGCAGGTCCTCT |  |  |  |
| Cytochrome P450, family 19, subfamily A, polypeptide 1a  (gonadal aromatase) | *cyp19a1a* | cyp19a1a-Fw | AGACAGCAGCCCAGGAGTTG | 1.99 | -2.98 | 0.99 |
|  |  | cyp19a1a-Rv | TGCAGTGAAGTTGATGTCCAGTT |  |  |  |
| Cytochrome P450, family 19, subfamily A, polypeptide 1b | *cyp19a1b* | cyp19a1b-Fw | CCCTTTTCAGCGCAGTGGTA | 2.01 | -3.29 | 0.94 |
| (brain aromatase) |  | cyp19a1b -Rv | CATTCGGCTTGTGGTGCTC |  |  |  |
| DmeI-CG10623 | *cg10623* | cg10623-Fw | AACTGGCCAAAGAGACGGTA | 2.26 | -2.78 | 0.99 |
|  |  | cg10623-Rv | CAGAGCCGTTGAGCAGAAAG |  |  |  |
| Doublesex- and mab-3- related transcription factor I | *dmrt1* | dmrt1-Fw | CCTTCACGCTACCCCACCTA | 2.20 | -2.92 | 0.97 |
|  |  | dmrt1-Rv | GTTGTTGTCGTCCAGGCTGA |  |  |  |
| Endoribonuclease Dicer | *dicer1* | dicer1-Fw | GCAGTACCGGAGCAGACTTA | 2.04 | -3.22 | 0.98 |
|  |  | dicer1-Rv | AGAGGACGGTGCTCAACATT |  |  |  |
| Euchromatic histone-lysine N-methyltransferase 2 | *ehmt2* | ehmt2-Fw | TGTGTTTGATGCATGGTGCT | 2.23 | -2.87 | 0.99 |
|  |  | ehmt2-Rv | AGACTTCATGTGTCAGGGCA |  |  |  |
| Extracellular matrix protein 1 | *ecm1* | ecm1-Fw | CAGAGCAGAGCACCCAGATA | 2.57 | -2.44 | 0.96 |
|  |  | ecm1-Rv | TCAGTCTCACAGCATGAAGGA |  |  |  |
| Glucocorticoid receptor | *nr3c1* | gr-Fw | CTTCCATCCAGCCCGTTGAT | 2.07 | -3.17 | 0.98 |
|  |  | gr-Rv | GTAGTGGAGGTCTGCGTCTG |  |  |  |
| Gonadotropin-releasing hormone | *gnrh* | gnRH-Fw | ACGCCCTGCAGAGTTTTAGG | 2.04 | -3.23 | 0.99 |
|  |  | gnRH-Rv | AGAAGCACGAGGTCCTGACA |  |  |  |
| Histone deacetylase 11 | *hdac11* | hdac11-Fw | ACAGCACTACTGGAAGCACT | 2.22 | -2.89 | 0.98 |
|  |  | hdac11-Rv | AGACGTTCTTCTCACCCGTT |  |  |  |
| HMG-box transcription factor SOX17 | *sox17* | sox17-Fw | CAAGAGACTGGCGCAGCAA | 2.25 | -2.84 | 0.98 |
|  |  | sox17-Rv | TTTCCACGATTTCCCCAACAT |  |  |  |
| Insulin-like growth factor I | *igf1* | igf1-Fw | TCCGTTTGTCACTTGTGTGAACT | 2.16 | -2.98 | 0.95 |
|  |  | igf1-Rv | AGGCAATCAAGCACCATGAA |  |  |  |
| Methyltransferase-like protein 22 | *mettl22* | metll22-Fw | CCAGGAAGTGGCTGAAGCTC | 2.18 | -2.63 | 0.97 |
|  |  | metll22-Rv | CTGCCTTGTGCTTCCTCTCC |  |  |  |
| Polycomb group ring finger 2 | *pcgf2* | pcgf2-Fw | CACTTCCACGGAATGAGACG | 2.07 | -3.16 | 0.99 |
|  |  | pcgf2-Rv | GCTGCACTGAGAGACAAACC |  |  |  |
| Prolactin | *prl* | prl-Fw | TATCCTGACCAGCGGATGTG | 2.10 | -3.09 | 0.96 |
|  |  | prl-Rv | ACGCTGCCACCATGTACAAC |  |  |  |
| Protein Jumonji | *jarid2a* | jarid2a-Fw | GGCTGAGCTCATGCATACAC | 2.01 | -3.30 | 0.98 |
|  |  | jarid2a-Rv | ACCAGACGTTCTTCACACCA |  |  |  |
| r18S | *r18S* | r18S-Fw | CCGCTTTGGTGACTCTAGATAACC | 2.09 | -3.13 | 0.99 |
|  |  | r18S-Rv | CAGAAAGTACCATCGAAAGTTGATAGG |  |  |  |
| Smoothelin 1 | *smtn1* | smtn1-Fw | CCTCAAGTTGGAAAGACGGC | 2.18 | -2.96 | 0.98 |
|  |  | smtn1-Rv | TGTTCTTATCTGCAGCGCAC |  |  |  |
| Steroidogenic acute regulatory protein | *star* | star-Fw | AGCAGAGGGGTGTTGTCAGA | 2.10 | -3.10 | 0.99 |
|  |  | star-Rv | TGGTTGGCAAAGTCCACCTG |  |  |  |
| Tescalcin | *tesc* | tesc-Fw | CAACATGGAGACCATCGCCC | 2.10 | -3.09 | 0.96 |
|  |  | tesc-Rv | TGAACATCCGTCCTCGGTCA |  |  |  |
| Troponin I | *tnnI* | tnnI-Fw | TGGTGTAGACAGCGGATACC | 2.38 | -2.65 | 0.96 |
|  |  | tnnI-Rv | TGCCAGTGCTTCATATCGGA |  |  |  |
| Vasa protein | *vasa* | vasa-Fw | CAGAAGCATGGCATTCCAATC | 2,24 | -2.86 | 0.99 |
|  |  | vasa-Rv | TGCAGAATAGGGAGCAGGAAA |  |  |  |
| WNT1 inducible signaling pathway protein 1 | *wisp1* | wnt1-Fw | CATGCGAGTGTCCGAAGTCC | 2.20 | -2.92 | 0.97 |
|  |  | wnt1-Rv | CGCACATCTTGCAGCAATCG |  |  |  |
