# Supplementary material for: Lasting effects of early exposure to temperature on the gonadal transcriptome at the time of sex differentiation in the European sea bass, a fish with mixed genetic and environmental sex determination
Source: BMC Genomics. 2015 Sep 4;16(1):679. doi: 10.1186/s12864-015-1862-0 (PMC4560065; doi:10.1186/s12864-015-1862-0)
Supplement: Additional file 2: Figure S1. — Photomicrographs of one-year-old European sea bass gonads. (A) LT females, (B) HT females, (C) LT males and (D) HT males. Scale bar = 50 μm. Figure S2. GO terms results and classification in two main categories of the upregulated genes in the HT group: A, molecular function (MF); and B, cell component (CC). Figure S3. GO terms results and classification in two main categories of the downregulated genes in the HT group: A, molecular function (MF); and B, cell component (CC). Figure S4. Diagram on European sea bass sex differentiation events, experimental design and sampling strategy. On a calibrated age scale, the bottom panel illustrates the main events related to gonadal sex differentiation. The middle panel depicts the low (LT) and high (HT) temperature periods, matching the thermosensitive period (TSP). The boxes indicate the sampling for transcriptomic analysis in relation to age and events of sex differentiation. The top panel highlights the two main samplings of the experiment and the type of the performed analyses. (DOCX 1693 kb) [file 12864_2015_1862_MOESM2_ESM.docx]

**Supplementary Figure 1**


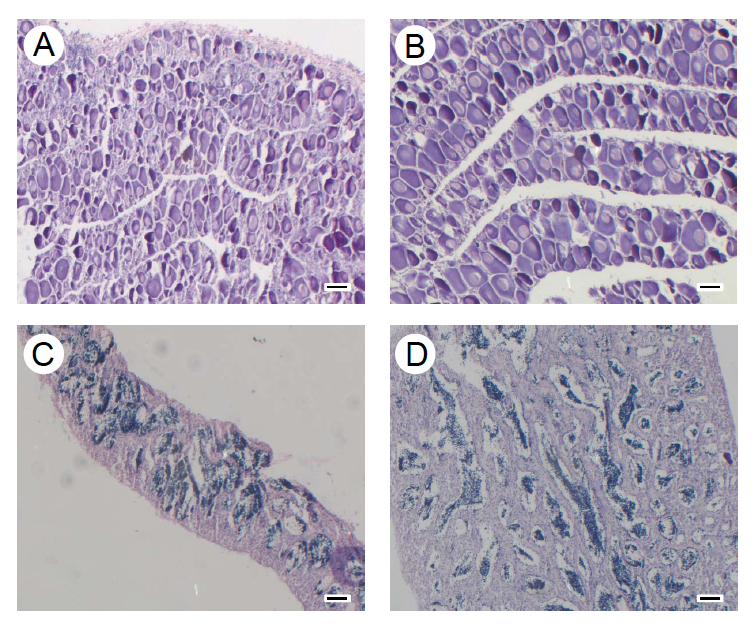


Supplementary Figure 1. Photomicrographs of cross sections of one-year-old European sea bass gonads. A) Low temperature females; B) High temperature females; C) Low temperature males; D) High temperature males. Scale bar = 50 µm.

**Supplementary Figure 2**


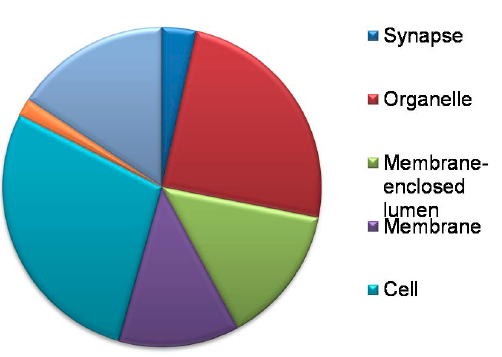

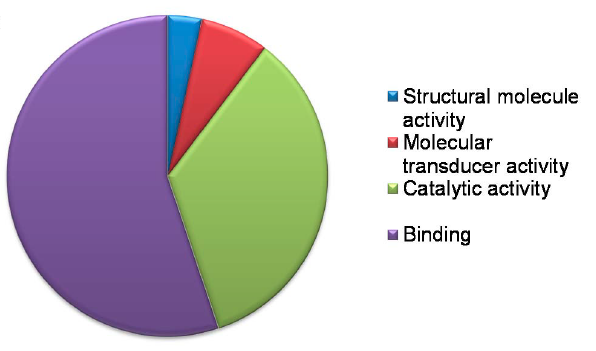


A

B

Supplementary Figure 2. GO terms results and classification in two main categories of the upregulated genes in the high temperature (HT) group. A) Molecular function (MF); B) Cell component (CC).

**Supplementary Figure 3**


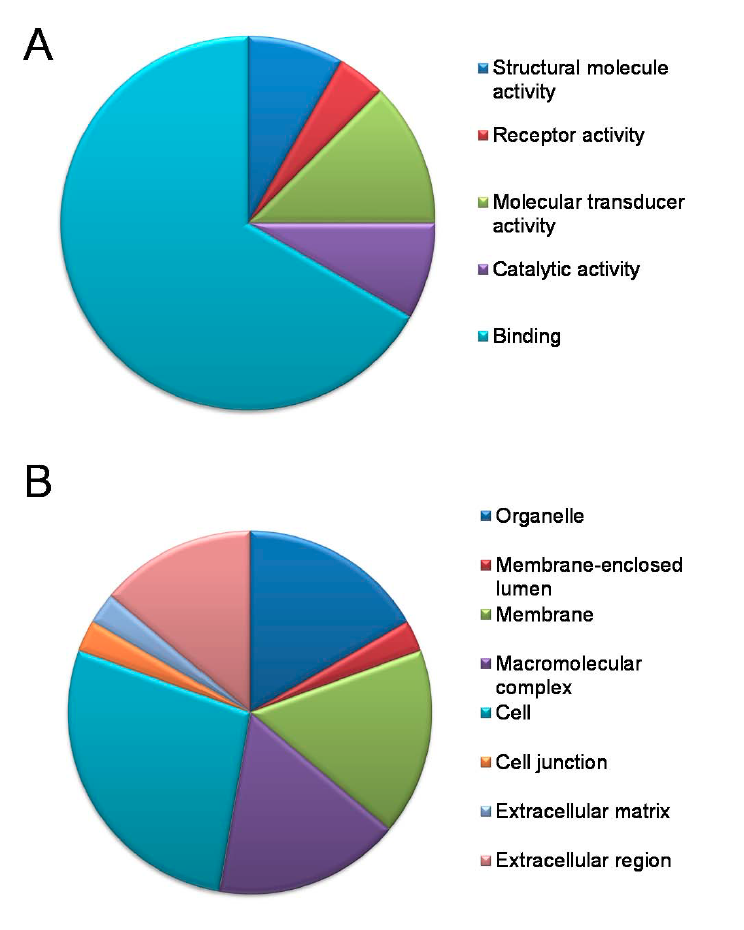


Supplementary Figure 3. GO terms results and classification in two main categories of the downregulated genes in the high temperature (HT) group. A) Molecular function (MF); B) cell component (CC).

**Supplementary Figure 4**

**
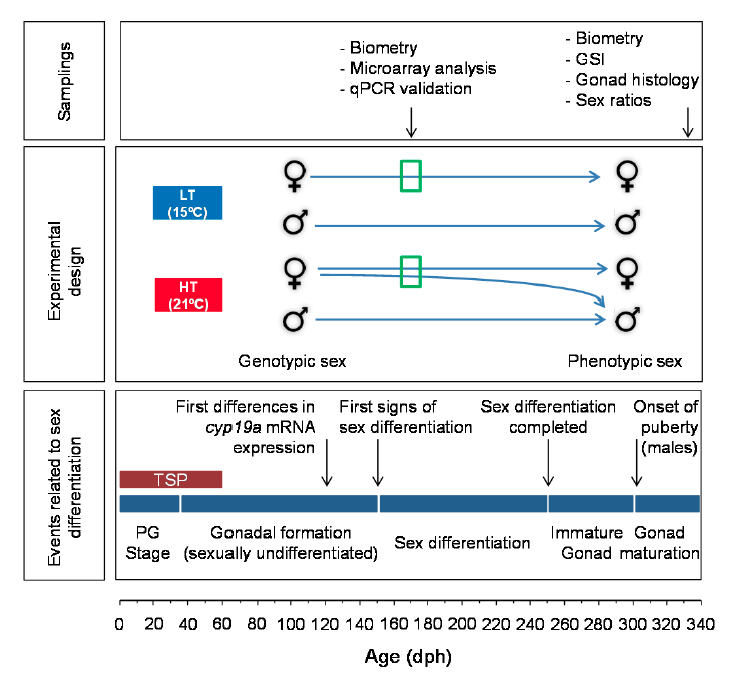
**

Supplementary Figure 4. Diagram on European sea bass sex differentiation events, experimental design and sampling strategy. On a calibrated age scale, the bottom panel illustrates the main events related to gonadal sex differentiation, from the pregonadal (PG) stage until the onset of puberty. The middle panel depicts the low (LT) and high (HT) temperature periods, within the thermosensitive period (TSP). The open green boxes indicate the sampling for transcriptomic analysis in relation to age and events of sex differentiation, meaning that in both groups females were targeted since fish with the highest *cyp19a1a* mRNA levels were chosen in each case. The top panel highlights the two main samplings of the experiment and the type of the analyses carried out in each one of them.
